# The serine-rich C-terminal tail of the *Listeria monocytogenes* secretion chaperone PrsA2 is critical for bacterial virulence and resistance to cell-wall active antibiotics

**DOI:** 10.1371/journal.ppat.1014676

**Published:** 2026-10-05

**Authors:** Allison H. Kumar, Charles Agbavor, Francis Alonzo 3rd, Laty A. Cahoon

**Affiliations:** 1 Department of Biological Sciences, University of Pittsburgh, Pittsburgh, Pennsylvania, United States of America; 2 Department of Microbiology and Immunology, University of Illinois at Chicago, Chicago, Illinois, United States of America; University of Colorado Anschutz Medical Campus, UNITED STATES OF AMERICA

## Abstract

Protein intrinsically disordered regions (IDRs) are characterized by their lack of defined stable tertiary structure and are capable of adopting multiple conformations for functional activity. In chaperone proteins, these flexible regions are often sites of condition specific protein-protein interactions. In *Listeria monocytogenes* (*Lm*), PrsA2 is a peptidyl prolyl isomerase (PPIase) chaperone that promotes pathogenesis through the maturation of secreted proteins including the pore forming toxin, listeriolysin O (LLO). PrsA2 is composed of a PPIase domain and foldase domain that are critical for secreted protein function. In addition, PrsA2 contains an uncharacterized unstructured region, the carboxyl-terminal tail (C-tail) consisting of 10-amino acid residues rich in serine. Here, we demonstrate that the PrsA2 C-tail and its serine residues are critical for *Lm* bacterial virulence in a mouse septicemic model. We trace this virulence defect to a reduction in secreted LLO activity exhibited by *Lm* PrsA2 C-tail bacterial mutants. Further, we use biophysical and biochemical assays to establish that the PrsA2 C-tail is required for interacting with and folding the LLO toxin. We then characterize the PrsA2 C-tail as a critical factor in *Lm* survival under stress conditions and highlight its crucial role against cell-wall targeting antibiotics. PrsA homologs are well-conserved in Gram-positive bacteria, and this work characterizes the *Lm* PrsA2 C-tail, demonstrating its contribution to bacterial virulence, chaperone function, and secreted protein activity.

## Author summary

Compared to eukaryotes, unstructured protein regions within bacterial proteomes are much less abundant, yet they are increasingly recognized as important for protein function and regulation. In this study, we characterize an unstructured C-terminal region of the peptidyl-prolyl isomerase chaperone PrsA2 from the Gram-positive food-borne pathogen *Listeria monocytogenes* (*Lm*). We demonstrate that this PrsA2 C-tail region is a key determinant of *Lm* virulence, enabling the activity of the major pore-forming toxin listeriolysin O. In addition, the PrsA2 C-tail is critical for overcoming cell-wall targeting antibiotics. Since PrsA homologs are well conserved in Gram-positive bacteria, our findings may be widely applicable to several Gram-positive pathogens.

## Introduction

Bacteria secrete proteins that provide structural and enzymatic functions which facilitate their ability to survive in diverse environments. In Gram-positive bacteria, approximately one-third of the proteome is secreted and translocated unfolded into the space between the cell membrane and cell wall [1–4]. This membrane-wall space is solvent exposed and contains a high density of negative charge associated with the cell-wall polymers, producing an environment that disfavors protein folding [3,5]. Therefore, Gram-positive bacteria encode secreted chaperones that are proposed to fold proteins and virulence factors within this harsh environment [4,6,7]. Chaperones typically bind to unfolded or loosely folded proteins to facilitate maturation, prevent aggregation, and maintain protein homeostasis [8–10]. However, the mechanisms that promote protein folding and activity post-membrane translocation remain unclear in Gram-positive bacteria.

Here, we use *Listeria monocytogenes* (*Lm*) as a model organism to study protein secretion, folding, and activation. *Lm* is ubiquitous in the environment, existing as a saprophyte, but can transition into an intracellular pathogen upon consumption by a mammalian host [11]. *Lm* can survive and grow over a wide range of environmental conditions, allowing the bacterium to overcome food preservation and safety barriers. *Lm* has been associated with various food-borne outbreaks, costing the food and health industries between 2.3 to 22 billion dollars per year [12]. In addition to its economic impact, *Lm* poses serious health risks to vulnerable populations including the immunocompromised, the elderly, pregnant women, and neonates [12–17]. Upon ingestion by a human host, *Lm* can transition into an intracellular pathogen where the fatality rate ranges from 20-50% in susceptible individuals [12,18–20].

A key underlying factor of *Lm* pathogenicity is the proper folding and secretion of virulence factors. During *Lm* infection, the transcriptional activator, PrfA, mediates the switch from saprophyte to life within a mammalian host [11,21,22]. Inside an infected host, PrfA promotes the expression of secreted virulence factors such as internalins (InlA and InlB) for *Lm* host cell attachment and invasion [11,21], listeriolysin O (LLO) and phospholipases (PlcA and PlcB) for *Lm* escape from the phagosome [21–27], and ActA for *Lm* cell-to-cell spread [11,21]. Altogether, secretion of these virulence factors enhances *Lm* survival within the host, and central to this phenomenon is the post-translocation secretion chaperone, PrsA2 [5,28,29]. The proposed contribution of PrsA2 is to facilitate the folding and maturation of several secreted virulence factors, providing *Lm* with a functional protein arsenal required for bacterial pathogenicity [5,28,30,31].

PrsA2 is a parvulin peptidyl prolyl isomerase (PPIase) chaperone that can catalyze the *cis*-to-*trans* isomerization of peptide bonds N-terminal to proline residues [28]. Structural comparison and phenotypic studies demonstrate that PrsA2 functions as a dimer and in addition to its PPIase domain, contains a foldase domain proposed to stabilize unfolded protein chains [31,32]. This chaperone also contains a serine-rich carboxyl-terminal region of unknown function: the PrsA2 C-tail. Primary sequence analysis confirms that serine-rich stretches of 10–15 residues at the C-terminal end are common in many PrsA homologs [33]. The C-tail of *Lm* PrsA2 is comprised of 10-amino acids that are polar, charged, and hydrophilic, while additional *in silico* structural analysis indicates this region exhibits characteristics of intrinsic disorder [34]. While 30–40% of eukaryotic proteins contain at least one intrinsically disordered region (IDR), only ~4% of bacterial proteins contain IDRs, therefore investigating these flexible regions is crucial to understanding protein function [35,36]. Unlike structured domains, these regions lack a stable tertiary structure and instead adopt dynamic conformations [35,37]. This structural flexibility enables diverse biological functions, including enhanced protein recognition and interactions with multiple targets [37]. Additionally in chaperone proteins, unstructured regions can facilitate physical interactions with client proteins [37,38]. Given that in *Lm,* at least 80 proteins from cell wall and supernatant factions rely on PrsA2 for maturation [31], we hypothesize that this unstructured C-terminal region of PrsA2 is required for client interactions and activity. Therefore, we investigated the role of the *Lm* PrsA2 C-tail in client protein interaction, folding, and regulation.

Here, we provide the first evidence that the serine-rich C-tail of *Lm* PrsA2 is critical for the regulation of an essential virulence determinant, the pore forming toxin, LLO. We demonstrate that absence of the PrsA2 C-tail reduces binding affinity to LLO, as well as the ability of PrsA2 to effectively fold LLO indicating that protein-protein interactions and protein folding requires the C-tail of PrsA2. Further, we show that the PrsA2 C-tail, in addition to its serine residues, are involved in virulence in a mouse model of infection. We also establish a role for the PrsA2 C-tail in bacterial physiology and resistance to cell-wall active antibiotics. Since serine-rich C-tails are highly conserved across distantly related PrsA homologs [29,33], characterizing their roles in virulence, protein-protein interactions, and protein folding will enhance our understanding of how molecular chaperones regulate virulence factors to promote bacterial pathogenesis.

## Results

### PrsA2 consists of a PPIase domain, foldase domain, and C-terminal tail

PrsA2 is a parvulin PPIase chaperone that is widely conserved in Gram-positive bacteria [4,29,33]. The PrsA2 full-length protein is composed of 293-amino acid residues and contains an N-terminal cysteine that is lipidated to the bacterial membrane upon signal peptide cleavage [39] (Fig 1A). Past structural comparison and phenotypic studies show that PrsA2 exists as a dimer and contains both PPIase and foldase domains [31,32] (Fig 1B & S1). The central PPIase domain, flanked by the N and C terminal domain have been previously characterized for roles in protein secretion and bacterial virulence [28,31]. In addition to these domains, PrsA2 contains a serine-rich carboxyl-terminus composed of 10-amino acid residues – SSSSDSDSSK, at positions 284–293, or the carboxyl-tail (C-tail) (Fig 1B). The enrichment of serine residues in the PrsA2 C-tail is widely conserved across many Gram-positive bacteria [29,40] (S2A,B Fig), and further computational analysis [34,40,41] indicates substantial conformational flexibility in this region (S3A Fig). This suggests that the PrsA2 C-tail adopts an undefined tertiary structure, with a variety of dynamic conformations [35]. We hypothesize that the enhanced flexibility of the PrsA2 C-tail provides multiple binding confirmations that facilitate client protein interactions.

**Fig 1:**
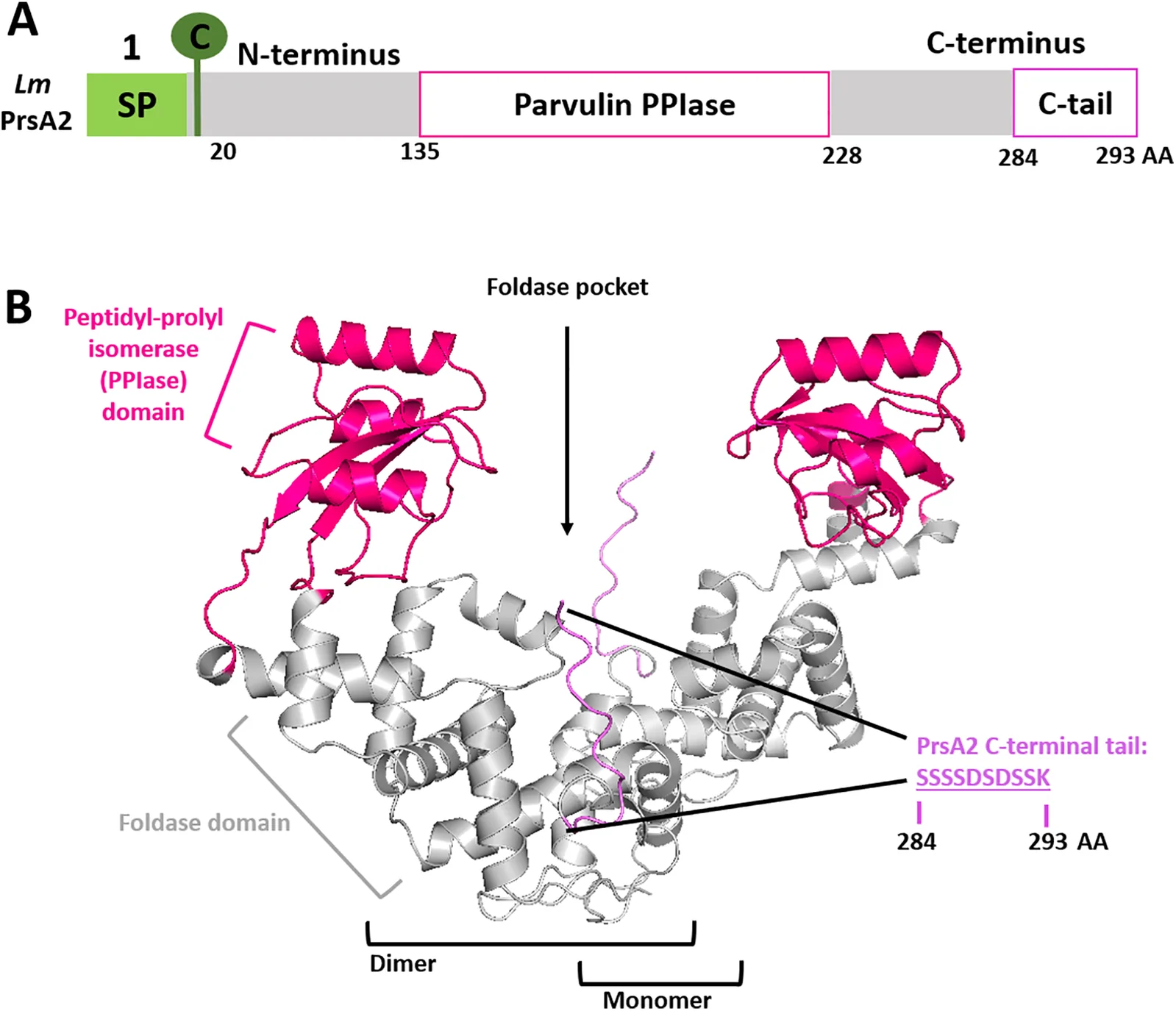
Domain organization and predicted structure of *Lm* PrsA2. A) PrsA2 is composed of 293 amino acids (AA), containing a signal peptide (SP), a lipid-modified N-terminal cysteine (C) at residue 21, and a parvulin PPIase domain located at position 135-228 residues. **B)** Cartoon of the PrsA2 dimeric structure modeled with Alphafold2 [76]. The PrsA2 PPIase domains and foldase domain and pocket are indicated. In addition, the sequence of the serine-rich C-terminal tail composed of 10-AA, spanning residues 284 to 293 is indicated.

### The PrsA2 C-tail and serine residues within the C-tail are critical for mouse bacterial virulence

It has been previously demonstrated that PrsA2 is essential for *Lm* virulence in mouse models of infection [30,42], such that when outbred Swiss-Webster mice were infected by tail-vein injection, the *prsA2* deletion mutant was severely attenuated compared to the wildtype strain [30]. Since we hypothesize that the PrsA2 C-tail facilitates client protein interactions, we asked whether the PrsA2 C-tail and/or the PrsA2 C-tail serine residues are also critical for mouse virulence. First, we generated a *Lm* PrsA2 C-tail deletion mutant (*prsA2*ΔC-tail) and a C-tail serine-to-alanine mutant (*prsA2*-StoA-tail). While the serine-to-alanine substitutions slightly decrease the predicted disorder propensity of the C-tail, this region is predicted to maintain substantial flexibility (S3B Fig). Then, we confirmed that *Lm* PrsA2 C-tail mutants exhibit wildtype levels of growth in broth culture and express similar levels of PrsA2 as compared to the wildtype strain (S4A-C Fig). Finally, we assessed these PrsA2 C-tail mutants for their ability to interfere with PrsA2 functions required for virulence in the mouse septicemic infection model (Fig 2). At 72 hours post intravenous infection, there is a significant reduction in bacterial burdens recovered from both livers and spleens of mice infected with the *prsA2*ΔC-tail and *prsA2*-StoA-tail mutants compared to wildtype bacteria (Fig 2), demonstrating that both the PrsA2 C-tail and the serine residues within the PrsA2 C-tail are critical for bacterial virulence. We note that the *prsA2*-StoA-tail mutant presents stronger defects in mouse virulence than the *prsA2*ΔC-tail mutant, suggesting that the tail serine residues may be critical for client interactions in the host and/or tail serine-to-alanine substitutions may hinder client interactions in the host.

**Fig 2:**
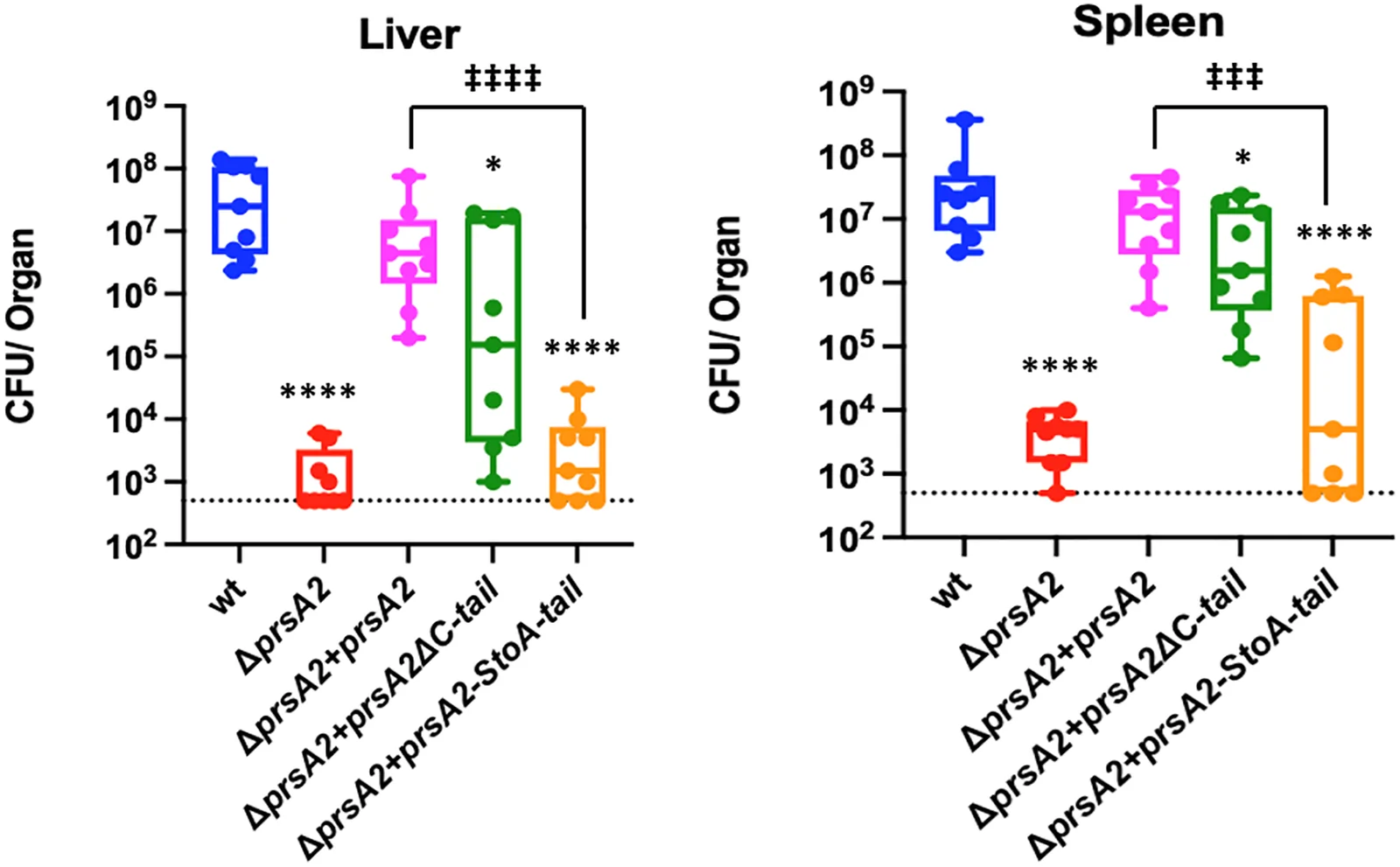
The C-tail of PrsA2 contributes to *Lm* bacterial virulence *in vivo.* Mice were infected with 2 × 10^4^ colony forming units (CFUs) of the indicated *Lm* bacterial strains by tail vein injection. At 72 hours post infection, livers and spleens were collected and plated for CFUs. Each point on the box plot represents one mouse and the dotted line indicates the limit of detection of 500 CFUs. Data were obtained from 9 mice. Statistical significance was calculated using a Mann-Whitney test. Statistical significance as compared to wildtype (wt) is indicated by ****P ≤ 0.0001 and *P ≤ 0.05. Statistically significant difference using a Mann-Whitney test compared to Δ*prsA2 + prsA2* are also indicated by ‡‡‡‡ *P* ≤ 0.0001 and ‡‡‡ *P* ≤ 0.001.

### PrsA2 C-tail mutants exhibit reduced patterns of intracellular growth in cell culture models

The mouse virulence defects associated with the *prsA2* C-tail mutants prompted us to examine these strains for intracellular growth in cell culture models. We first used three macrophage cell-types, RAW264.7, J774, and primary bone-marrow derived macrophages to assess the contribution of the PrsA2 C-tail to intracellular growth and replication (Fig 3–4). The Δ*prsA2*, *prsA2*ΔC-tail, and *prsA2*-StoA-tail strains exhibit a pronounced defect in intracellular growth over an 8 hour time course as compared to wildtype and the *prsA*2 complemented strain in RAW264.7, J774, and bone-marrow derived macrophages infected with a multiplicity of infection (MOI) of 0.8, 0.3, and 0.3, respectively (Fig 3A&C&E, S5A-C Fig). We also observe these defects after normalization for bacterial uptake at 1 hour post-infection (S6A&B Fig). At 8 hours post-infection, the number of mutant bacteria was significantly less than wildtype (Fig 3B&D&F), highlighting a replication defect within host cells. Since the *prsA2* C-tail mutants showed defects in macrophage intracellular growth at 8 hours, we next asked whether this defect was also present at early time points using an MOI range of 0.1, 1, and 10. We infected RAW264.7 cells with *Lm* wildtype*,* Δ*prsA2*, *prsA2* C-tail mutants and the *prsA2* complemented strain and measured the amount of intracellular bacteria at 2 hours post-infection (Fig 4). The Δ*prsA2*, *prsA2*ΔC-tail, and *prsA2*-StoA-tail mutants all show reduced viable bacteria indicating a significant growth defect early during infection of RAW264.7 macrophages (Fig 4).

**Fig 3:**
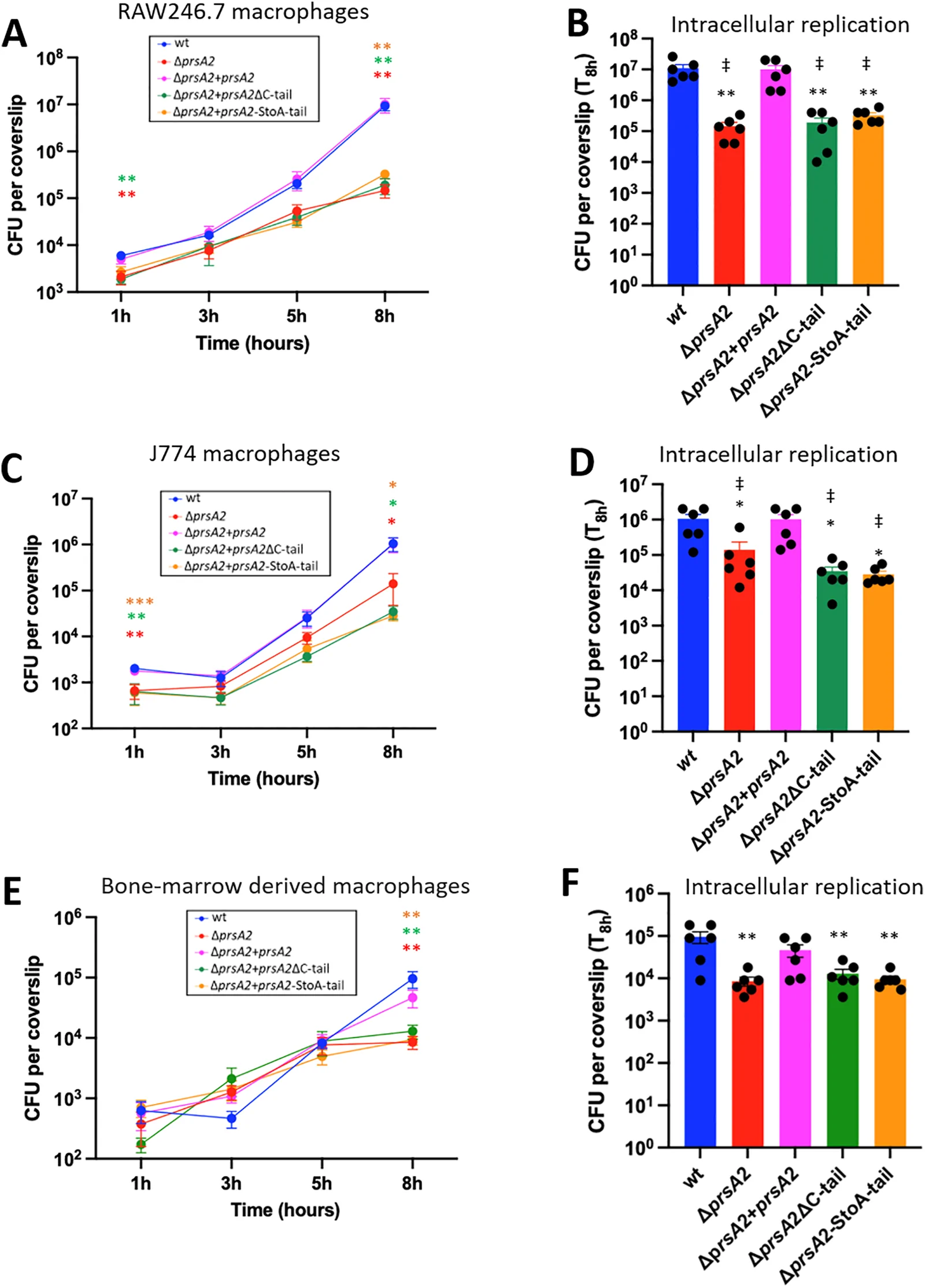
PrsA2 C-tail mutants exhibit intracellular growth defects in phagocytic cell culture models. Intracellular growth of the *Lm* wildtype (wt) and serine tail mutants was assessed in RAW264.7 macrophages **(A)**, J774 macrophages **(C)**, and bone-marrow derived macrophages (BMMØ) **(E)**. Cells were infected with the *Lm* wt, Δ*prsA2,* Δ*prsA2 + prsA2,* Δ*prsA2* + Δ*prsA2*ΔC-tail, or Δ*prsA2* + Δ*prsA2-*StoA-tail strains at a multiplicity of infection (MOI) of 0.8, 0.3 and 0.3 for RAW264.7 and J774 macrophages, and BMM, respectively. After 30 minutes post-infection, cells were washed with PBS, followed by the addition of 10 µg/ml gentamicin at 1 hour post-infection to kill extracellular bacteria while allowing enough time for bacterial internalization. *Lm* intracellular replication in RAW264.7 macrophages **(B)**, J774 macrophages **(D)**, and BMM **(F)** was determined as the number of CFUs at the 8 hour time point for each indicated strain. Asterisks (*) and daggers (‡) indicate statistical significance of *P* ≤ 0.001, *P* ≤ 0.01, and *P* ≤ 0.05 using a One-way ANOVA with Tukey’s multiple comparisons test compared to the wt and Δ*prsA2 + prsA2* strain*,* respectively and the error bars represent the standard error of the mean. The data shown are representative of two independent experiments conducted in triplicate.

**Fig 4:**
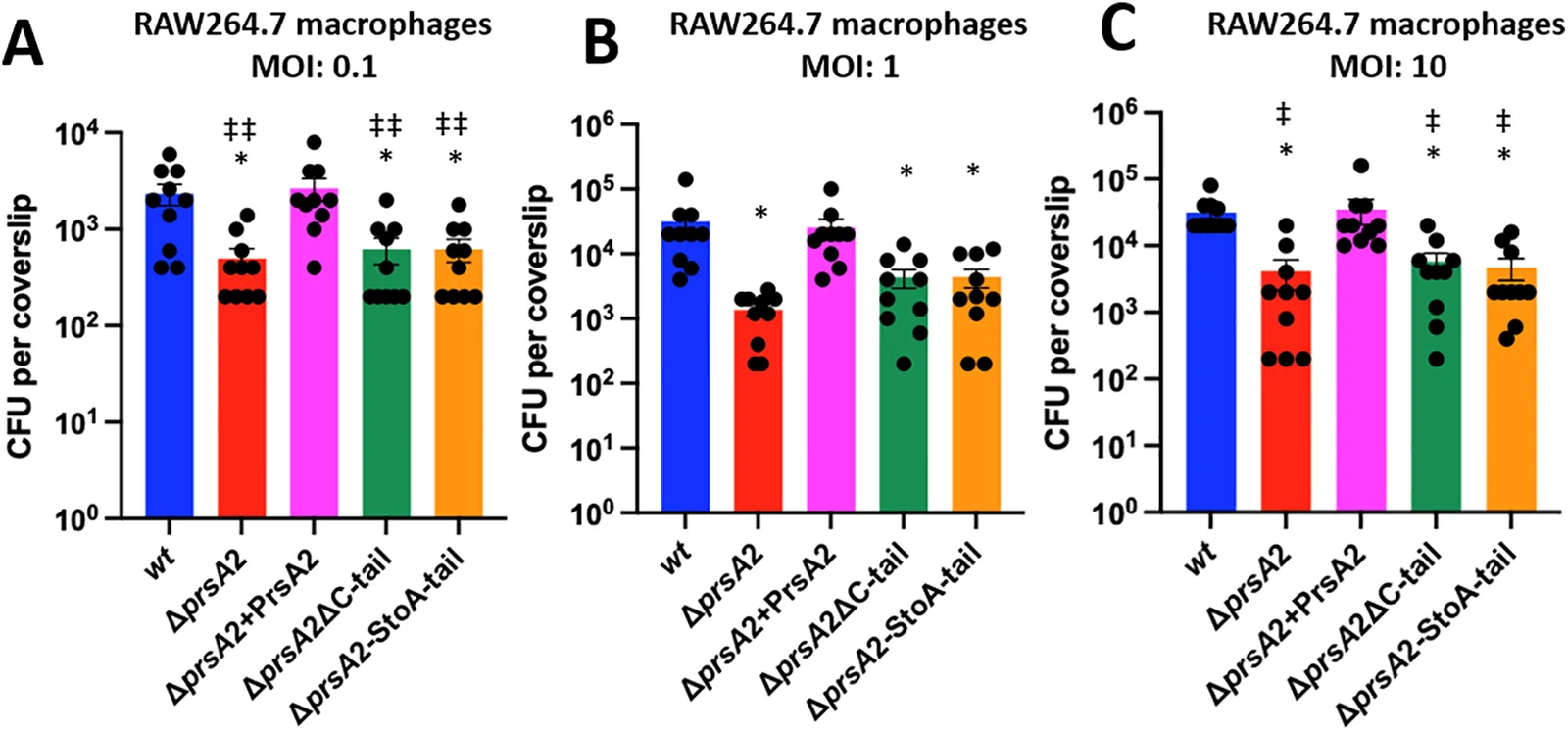
PrsA2 C-tail mutants demonstrate early reduced viability in RAW264.7 macrophages. RAW264.7 macrophages were infected with *Lm* wt, Δ*prsA2,* Δ*prsA2 + prsA2,* Δ*prsA2* + Δ*prsA2*ΔC-tail, or Δ*prsA2* + Δ*prsA2-*StoA-tail strains with a MOI of 0.1 **(A)**, 1 **(B)**, and 10 **(C)** to assess early viability. After 30 minutes post-infection, the cells were washed with PBS followed by the addition of 10 µg/ml gentamicin at 1 hour post-infection to kill extracellular bacteria. At 2 hours post-infection, the CFUs for each indicated strain was determined. Asterisks (*) and daggers (‡) indicate statistical significance of *P* ≤ 0.05 and *P* ≤ 0.01 using a One-way ANOVA with Tukey’s multiple comparisons test compared to the wt and Δ*prsA2 + prsA2* strain*,* respectively and the error bars represent the standard error of the mean. The data shown are representative of two independent studies combined, each containing five biological replicates.

To test whether the *prsA2* C-tail mutants also exhibit intracellular growth defects in non-phagocytic cells, we used human colon derived Caco-2 cells and infected with *Lm* strains at an MOI of 50 (Fig 5A–C, S5D). We observe reduced colony forming units (CFUs) recovered from the Δ*prsA2*, *prsA2* C-tail, and *prsA2*-StoA-tail mutants as compared to the wildtype and *prsA*2 complemented strain over a 7 hour time course (Fig 5A–C). Together, these data suggest that the *prs*A2 C-tail mutants have impaired intracellular growth, potentially indicating a defect in internalin-mediated entry in addition to phagosome escape.

**Fig 5:**
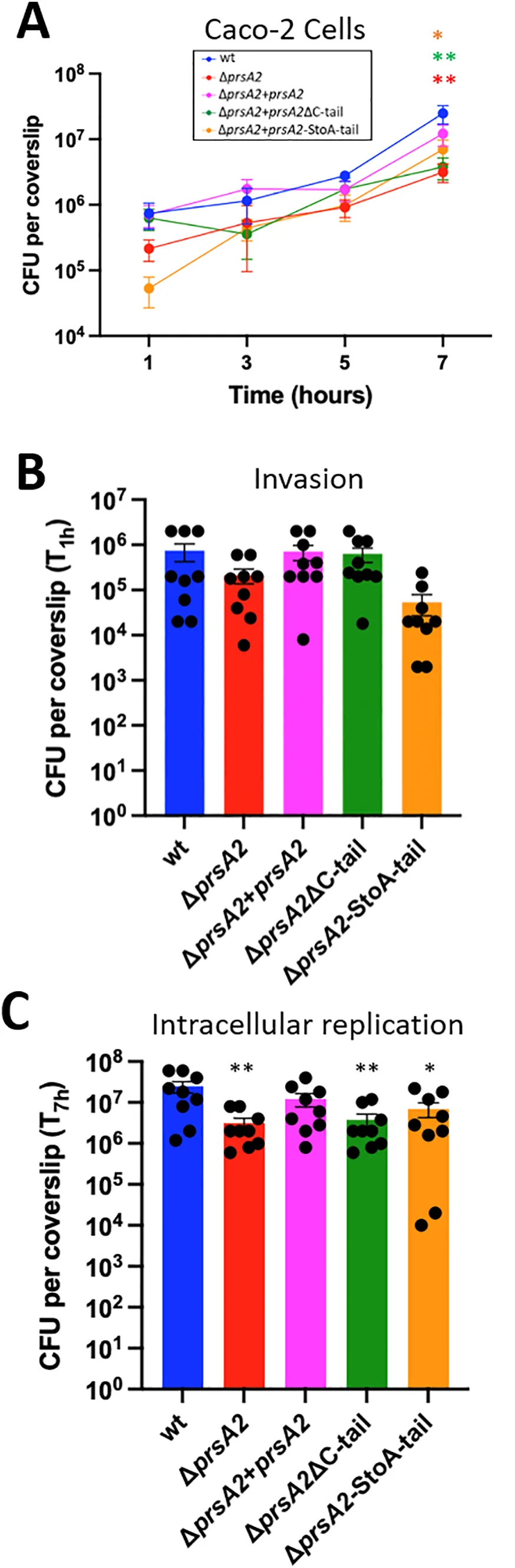
PrsA2 C-tail mutants exhibit intracellular growth defects in non-phagocytic cells. Intracellular growth of *Lm* wt and serine tail mutants was assessed in CaCo-2 human colon adenocarcinoma cells. The cells were infected with *Lm* wt, Δ*prsA2,* Δ*prsA2 + prsA2,* Δ*prsA2 +* Δ*prsA2*ΔC-tail, or Δ*prsA2 +* Δ*prsA2-*StoA-tail strains at a MOI of 50 and intracellular growth was measured over time **(A)**. At 1 hour post-infection, the cells were washed with PBS followed by the addition of 10 µg/ml gentamicin to kill extracellular bacteria. For each indicated strain, the initial invasion **(B)** and intracellular replication **(C)** was determined as the number of CFUs at the 1 hour and 7 hour time points, respectively. Asterisks indicate statistical significance of *P* ≤ 0.01, and *P* ≤ 0.05 using a One-way ANOVA with Tukey’s multiple comparisons test compared to the wt and the error bars represent the standard error of the mean. The data shown are representative of three independent experiments conducted in triplicate.

### The PrsA2 C-tail is critical for secreted listeriolysin O activity

Since the PrsA2 C-tail mutants exhibit virulence defects in mice, and defects in intracellular growth in both macrophage and epithelial cell lines, we investigated whether the PrsA2 C-tail contributes to additional virulence-associated phenotypes including listeriolysin O (LLO) activity, broad range phospholipase (PC-PLC) activity, and cell-to-cell spread. LLO and PC-PLC enzymes are secreted virulence factors that promote *Lm* phagosome escape [23–26] and the absence of *prsA*2 is associated with reduced LLO hemolytic activity, reduced phospholipase activity, and reduced cell-to-cell spread [30,32]. Therefore, we asked if the PrsA2 C-tail also contributes to these phenotypes. *Lm* strains expressing the *prsA*2ΔC-tail and the *prsA*2-StoA-tail exhibit modest defects in cell-to-cell spread (S7A Fig); and we found that the PrsA2 C-tail is not required for secreted broad range phospholipase activity (S7B Fig).

Next, we tested for secreted LLO activity by measuring the ability of *Lm* bacterial supernatants to lyse sheep’s red blood cells (RBCs). We determined that at pH 5.0, when LLO demonstrates near optimal activity [43], the *prsA*2ΔC-tail and the *prsA*2-StoA-tail strains exhibit a reduction in activity at ~42% and ~38% of the wildtype strain, respectively (Table 1), suggesting that the PrsA2 C-tail is critical for LLO maturation. Because chaperones tend to bind to loosely folded proteins [10], we also tested for secreted hemolytic activity at pH 6.5, where LLO is partially folded [31]. At these conditions, the *prsA*2ΔC-tail and the *prsA*2-StoA-tail mutants also show decreased hemolytic activity as compared to the wildtype strain (Table 1). These results indicate that the PrsA2 C-tail as well as serine residues within the C-tail are crucial for the secretion of functional LLO which may contribute to the defects observed in intracellular growth and cell-to-cell spread, in addition to the reduction in mouse virulence.

**Table 1:** The PrsA2 C-tail is required for secreted LLO activity at pH 5.0 and pH 6.5.

Hemolytic Units (AU)				
Strain	pH 5.0		pH 6.5	
	**Mean**	**FR**	**Mean**	**FR**
Wildtype	100 ± 0.0	–	100 ± 0.0	–
Δ*prsA2*	20.42 ± 4.27****	0.80	58.33 ± 12.91****	0.42
Δ*prsA2* + *prsA2*	105 ± 27.39	–	100 ± 0.0	–
Δ*prsA2* + *prsA2*ΔC-tail	41.67 ± 11.79***	0.58	66.67 ± 12.91****	0.33
Δ*prsA2* + *prsA2*-StoA-tail	38.33 ± 11.18***	0.61	87.5 ± 13.70*	0.13

Statistical significance is indicated by ****P ≤ 0.0001, ***P ≤ 0.001, and *P ≤ 0.05, using an unpaired Student’s two-tailed T-test compared to the wildtype strain. Data are represented as the mean ± the standard deviation. Fold reduction (FR) is indicated compared to the wildtype strain. Hemolysis by the wildtype strain was set to 1, and fold reduction in hemolysis for the mutant strains were calculated by dividing their means by 100 and subtracting 1. FR closer to 1 indicates impaired hemolysis.

### The PrsA2 C-tail is required for optimal binding to LLO

Recently, we demonstrated that PrsA2 interacts directly with LLO under conditions where LLO is partially folded at pH 6.5 [31]. To test whether the PrsA2 C-tail is required for this direct interaction with LLO, we used biophysical approaches, microscale thermophoresis (MST) and isothermal titration calorimetry (ITC). First, we confirmed that the secondary structures of the recombinant PrsA2 C-tail mutant proteins fold similarly to wildtype PrsA2 using circular dichroism spectroscopy (S8A Fig). Since PrsA2 acts as a dimer [31,32], we next verified that the recombinant PrsA2 C-tail mutant proteins form dimers using *in vitro* crosslinking experiments (S8B Fig). Additionally, in their native state, PrsA2 and the PrsA2 C-tail mutant proteins form dimers (S9 Fig).

For MST experiments, recombinant wildtype PrsA2, PrsA2ΔC-tail, and PrsA2-StoA-tail mutants were labeled with N-hydroxysuccinimide (NHS) dye and titrated against unlabeled LLO at pH 6.5. We have previously shown that PrsA2 physically interacts with LLO at nanomolar affinity [31], which is consistent with our results indicating that PrsA2 interacts with LLO with a *K*_*D*_ of ~17 nM (Fig 6A and Table 2). Since PrsA2 directly binds to LLO, we were interested in determining whether the C-tail region of PrsA2 affects its ability to interact with LLO. Therefore, we used MST to test the interaction between the recombinant PrsA2ΔC-tail mutant and LLO at pH 6.5. Strikingly, there is a 35-fold reduction in the binding affinity when compared to wildtype PrsA2, as the PrsA2ΔC-tail mutant interacts with LLO with a *K*_*D*_ of ~600 nM (Fig 6B and Table 2). Next, we asked whether the serine residues within the PrsA2 C-tail are required for this interaction with LLO. Therefore, we used MST to test the association between the recombinant PrsA2-StoA-tail mutant and LLO at pH 6.5. Surprisingly, the binding affinity of the PrsA2-StoA-tail mutant to LLO is 2.4-fold stronger when compared to wildtype PrsA2 (Fig 6C and Table 2).

**Table 2:** Thermodynamic parameters of the interactions between PrsA2 or PrsA2 C-tail mutants with LLO.

	MST	ITC^*b*^						
Strains	K_D_ (nM)^*a*^	K_D_ (nM)	n	ΔH (kJ/mol)	ΔG (kJ/mol)	-TΔS (kJ/mol)	ΔS (J/mol^.^K)	Fold difference
Wild type	17 [2, 53]	27.79 ± 1.41	2.40 ± 0.10	-80.79 ± 11.28	-43.26 ± 0.49	37.53 ± 12.30	-125.89 ± 37.35	–
PrsA2ΔC-tail	600 [200, 1700]	668.40 ± 40.94****	1.47 ± 0.07	-54.23 ± 4.82	-35.25 ± 0.15****	18.98 ± 4.95	-63.67 ± 16.60	-24.05
PrsA2-StoA-tail	7 [2, 17]	9.41 ± 4.99	2.94 ± 0.07	-97.09 ± 2.96	-46.04 ± 0.29*	51.04 ± 2.63	-171.2 ± 8.84	+2.95

***^a^*** Represents the dissociation constant (*K*_*D*_) from all MST experiments. Using PALMIST, a 1:1 *K*_*D*_ fitting parameter with a 95% confidence interval (lower and upper values in parentheses) was applied, as determined by “error-surface projection” (ESP).

***^b^*** Denotes the ITC thermograms of PrsA2, PrsA2ΔC-tail, and PrsA2-StoA-tail titrated into recombinant LLO. Fold difference was calculated by dividing the *K*_*D*_ of the PrsA2 C-tail mutant by wildtype PrsA2. The fold difference indicated by a minus sign (-) denotes reduced affinity while a plus sign (+) denotes enhanced affinity compared to wildtype PrsA2. The *K*_*D*_ is the dissociation constant, △*H* is the enthalpy, *n* is the stoichiometry, △*G* is the Gibbs free energy, and △*S* is the entropy of the interactions. An independent fitting model was applied to the ITC data using a 95% confidence interval and was used to calculate the error of each parameter. All experiments were performed three independent times with at least two separate batches of protein. Statistical significance is indicated by ****P ≤ 0.0001 and *P ≤ 0.05 using a One-way ANOVA with Tukey’s multiple comparisons compared to wildtype PrsA2.

**Fig 6:**
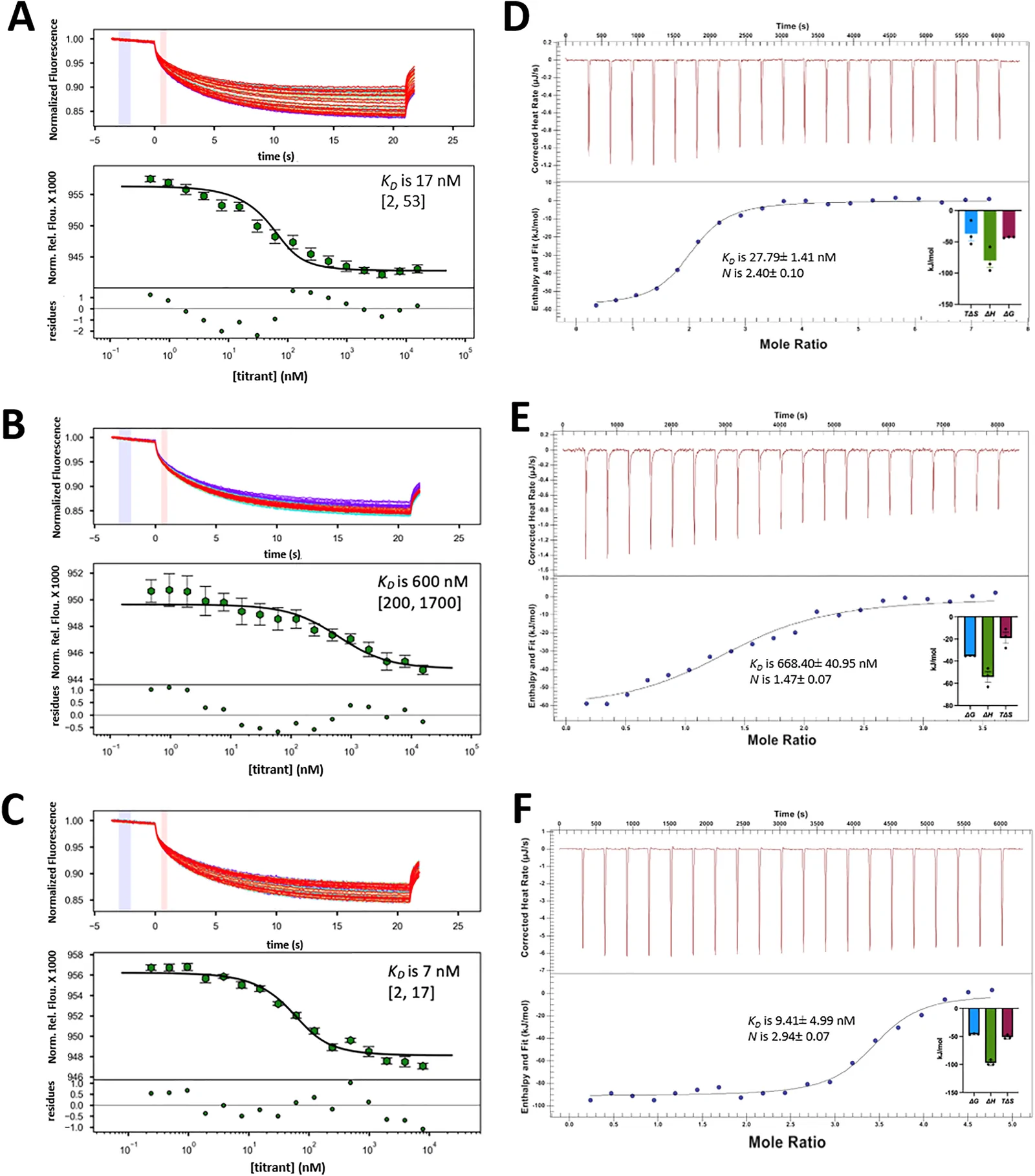
The PrsA2 C-tail is critical for interaction with LLO. Microscale thermophoresis (MST) of recombinant PrsA2 (14.2 µM) labeled with NHS dye titrated against unlabeled LLO (31.4 µM) **(A)**, recombinant PrsA2ΔC-tail (17.4 µM) labeled with NHS dye titrated against unlabeled LLO (31.4 µM) **(B)**, and recombinant PrsA2-StoA-tail (6.1 µM) labeled with NHS dye titrated against unlabeled LLO (15.7 µM) **(C)** is shown. The thermophoretic time traces are depicted in the top panels, where the blue and pink regions represent the time spans used to acquire the fluorescence cold (*F*_c_) and hot (*F*_h_) regions. The binding curve is represented in the middle panels, where the data points were averaged, the fluorescence was normalized, and a 1:1 *K*_*D*_ fitting parameter with a 95% confidence interval was applied. The bottom panels depict the residuals between the data and the fit. PALMIST [74,75] was used to analyze the results and error bars represent the standard deviation of three or more independent replicates. Isothermal titration calorimetry (ITC) thermograms of recombinant PrsA2 (23.3 µM) titrated into LLO (0.67 µM) **(D)**, recombinant PrsA2ΔC-tail (126 µM) titrated into LLO (0.5 µM) **(E)**, and recombinant PrsA2-StoA-tail (26.5 µM) titrated into LLO (0.75 µM) **(F)** at pH 6.5 and 25°C. The binding isotherm (top) and heat integration (bottom) are provided. An independent model was used to fit the titration curve and plot the thermodynamic parameters where the *K*_D_ and *N* are indicated. The inset bar graph shows the Δ*G,* Δ*H,* and –*T*Δ*S* for three independent experiments. Data represents three independent experiments with at least two separate batches of protein.

Further, we tested if PrsA2 and the C-tail mutants can interact with LLO under physiological conditions resembling the phagosome and the cytosol. Using MST at a pH 5.5, the approximate pH of the phagosome, we show that PrsA2, PrsA2ΔC-tail, and PrsA2-StoA-tail interacts with LLO with *K*_*D*_ values of 130, 1400, and 60 nM, respectively (Fig 7A–C). The weaker binding affinities suggest that PrsA2 and the C-tail mutants are rapidly interacting, folding, and releasing functional LLO needed to lyse the phagosomal membrane. In the context of the cytosol, we performed MST at pH 7.4 and demonstrate that PrsA2, PrsA2ΔC-tail, and PrsA2-StoA-tail interacts with LLO with *K*_*D*_ values of 17, 140, and 9 nM, respectively (Fig 7D–F). The tighter association suggests that PrsA2 and the C-tail mutants are regulating the activity and release of LLO, where its release is important for infection. To further ensure accurate measurements of native protein-protein interactions, the 6-His tag was removed from purified recombinant PrsA2, PrsA2ΔC-tail mutant, and PrsA2-StoA-tail mutant proteins prior to MST experiments. The resulting *K*_*D*_ values are within the range of the tagged constructs, indicating that the 6-His tag has minimal effects on the interaction of PrsA2 and the PrsA2 tail mutants with LLO (S10A-C Fig). Altogether, the MST data highlights that the C-tail region of PrsA2 is critical for interacting with LLO.

**Fig 7:**
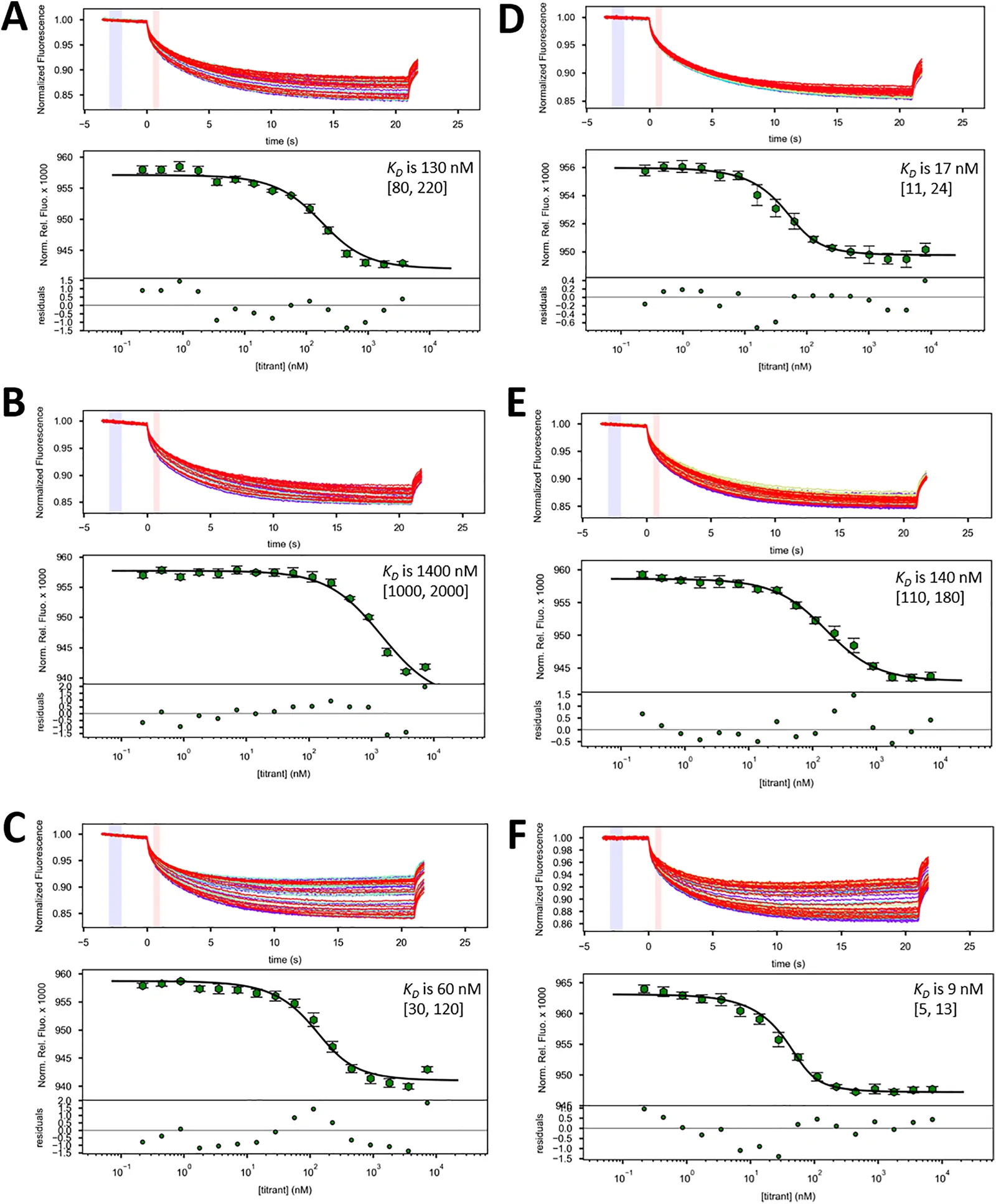
The interaction of PrsA2 and PrsA2 C-tail mutants in the context of the phagosome and cytosol. MST of recombinant PrsA2 (10.9 µM) labeled with NHS dye titrated against unlabeled LLO pH 5.5 (14.5 µM) **(A)**, recombinant PrsA2ΔC-tail (15.3 µM) labeled with NHS dye titrated against unlabeled LLO pH 5.5 (14.5 µM) **(B)**, recombinant PrsA2-StoA-tail (9.6 µM) labeled with NHS dye titrated against unlabeled LLO pH 5.5 (14.5 µM) **(C)**, recombinant PrsA2 (7.7 µM) labeled with NHS dye titrated against unlabeled LLO pH 7.4 (14.2 µM) **(D)**, recombinant PrsA2ΔC-tail (7.5 µM) labeled with NHS dye titrated against unlabeled LLO pH 7.4 (14.2 µM) **(E)**, and recombinant PrsA2-StoA-tail (1.4 µM) labeled with NHS dye titrated against unlabeled LLO pH 7.4 (14.2 µM) **(F)** is shown. The top panels represent the thermophoretic time traces, the middle panels highlight the binding curve, and the bottom panel bottom panels show the residuals between the data and the fit. PALMIST [74,75]was used to analyze the data and error bars represent the standard deviation of three or more independent replicates.

To complement our MST analysis and further define the binding parameters of recombinant PrsA2 C-tail mutants with recombinant LLO, we used ITC. As a control experiment for ITC, using recombinant wildtype PrsA2, PrsA2ΔC-tail, PrsA2-StoA-tail, and LLO proteins, we tested for binding interactions of each protein into buffer alone (S11A-C Fig). Then at pH 6.5, we demonstrate that PrsA2 physically interacts with LLO with *K*_*D*_ of 27.79 ± 1.41 nM and stoichiometry of ~2:1, highlighting that one PrsA2 dimer interacts with a monomer of LLO (Fig 6D, S12A-B, & Table 2). Consistent with our MST data, we show that the binding affinity between the PrsA2ΔC-tail mutant and LLO is reduced compared to wildtype PrsA2, as the *K*_*D*_ of this interaction is 668.40 ± 40.94 nM (Fig 6E, S13A-B, & Table 2). There is a 24-fold reduction in binding affinity with a stoichiometry of ~1.5:1 which highlights the inefficiency of the interaction of the PrsA2ΔC-tail mutant with LLO (Fig 6E, S13A-B, & Table 2).

Subsequently, using ITC, we tested the interaction between the PrsA2-StoA-tail mutant and LLO which demonstrated enhanced binding compared to wildtype PrsA2 (Fig 6F, S14A-B, & Table 2). The PrsA2-StoA-tail mutant interacts with LLO with a *K*_*D*_ of 9.41 ± 4.99 nM, similar to our MST values (Fig 6F, S14A-B, & Table 2). This enhanced binding between PrsA2-StoA-tail mutant and LLO suggests that the serine residues within the PrsA2 C-tail may contribute to LLO release. We speculate that increasing the hydrophobicity of the PrsA2 C-tail by mutating the polar serine residues to hydrophobic alanine residues may enhance binding to LLO [44]. This is also reflected in the stoichiometry (*N* = 2.94), as multiple PrsA2-StoA-tail molecules are interacting with a single LLO molecule compared to wildtype. In addition, the binding between PrsA2 or the PrsA2 C-tail mutants with LLO represent interactions that are both favorable and spontaneous (Table 2). Taken together, both the MST and ITC data highlight that the PrsA2 C-tail and the serine residues within the PrsA2 C-tail are critical for physical interactions with LLO.

### The serine-rich C-tail of PrsA2 is critical for LLO activation

Since PrsA2 C-tail mutants are capable of interacting with LLO, we asked whether these chaperone mutants also have the ability to effectively fold and activate denatured LLO at pH 6.5. Therefore, we used our recently developed chaperone-assisted protein folding assay [31]. This assay measures hemolysis as a readout for folded LLO activity, where purified recombinant LLO is chemically denatured with 8 M urea, diluted 20-fold, and added to increasing concentrations (0.01 µM - 2 µM) of PrsA2 chaperone. Then functional LLO activity is determined as the ability to lyse sheep’s red blood cells. We measured the activity of denatured LLO incubated with wildtype PrsA2, PrsA2ΔC-tail, and PrsA2-StoA-tail chaperones after 45 minutes (S15 Fig) and 90 minutes (Fig 8). When LLO is incubated for a longer period of time (90 minutes) with the chaperones, we observe that PrsA2 and mutant chaperones all confer varying degrees of LLO hemolytic activity when compared to the denatured LLO alone control (Fig 8). Whereas when LLO activity was assessed at 45 minutes, only wildtype PrsA2 and the PrsA2-StoA-tail mutant produced significant differences in activity for most concentrations tested when compared to the denatured LLO alone control (S15 Fig). When comparing the hemolytic activity of LLO incubated with PrsA2 or PrsA2 C-tail mutants, we observe that the PrsA2ΔC-tail mutant is less efficient at folding denatured LLO than wildtype PrsA2 (Fig 8). The PrsA2-StoA-tail mutant exhibits an intermediate phenotype, where it is less efficient at folding LLO than wildtype PrsA2, but more efficient than the PrsA2ΔC-tail mutant (Fig 8). Altogether, these data indicate that the folding and activation of denatured LLO is facilitated by the PrsA2 C-tail and serine residues within the PrsA2 C-tail.

**Fig 8:**
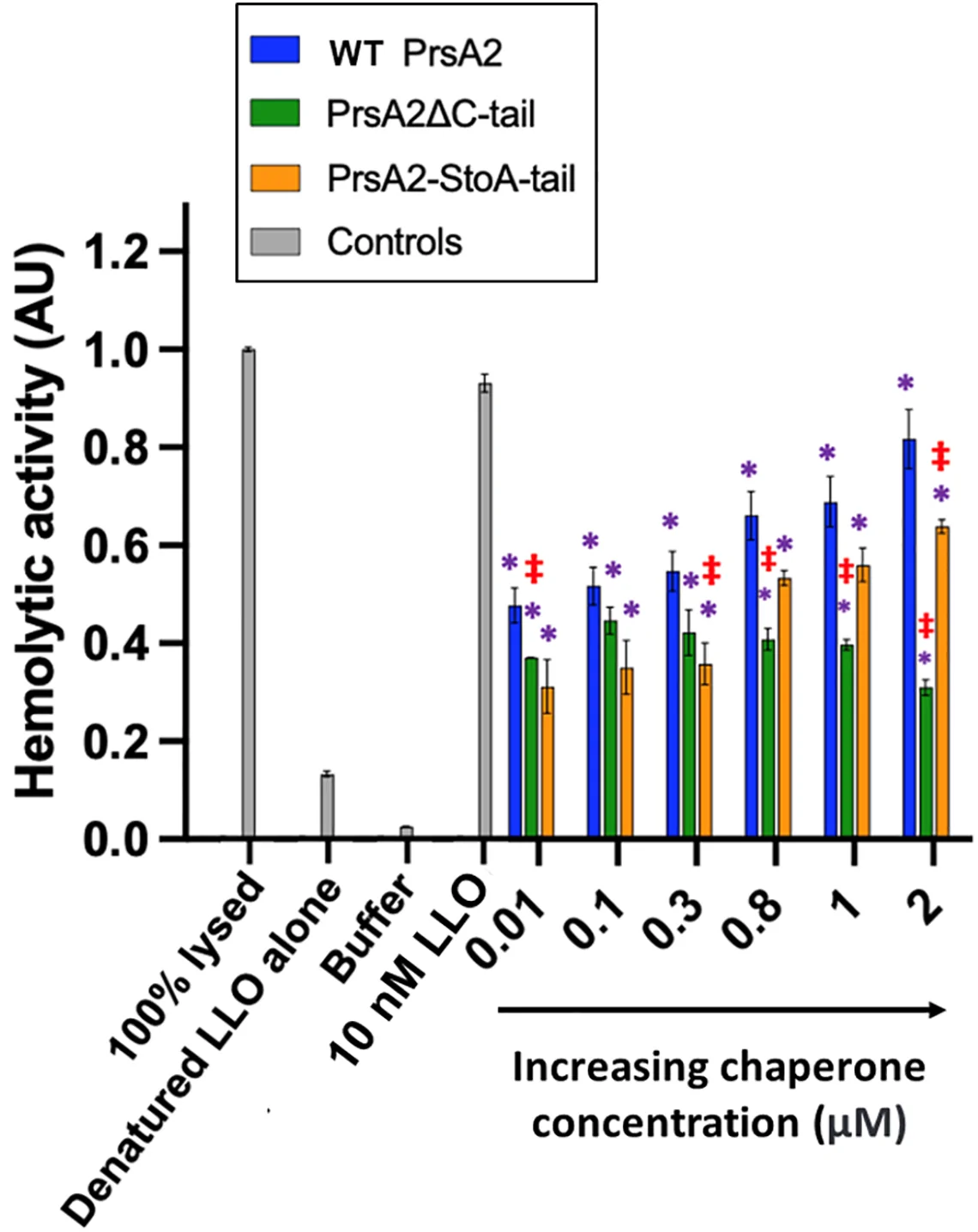
The C-tail of PrsA2 is critical for activation of denatured LLO. Recombinant PrsA2, PrsA2ΔC-tail, and PrsA2-StoA-tail were assessed for the ability to fold chemically denatured LLO using a chaperone-assisted protein folding assay at pH 6.5. First, buffer containing 8 M urea was used to denature LLO, then this denatured LLO was diluted 20-fold in PBS pH 6.5, subsequently the hemolytic activity of LLO was measured after 90 minutes to determine functional LLO activity in solution in the presence of increasing concentrations of PrsA2 or PrsA2 C-tail mutants (ranging from 0.01-2.0 µM) at pH 6.5. As controls, 100% sheep’s red blood cell (RBC) lysis was measured, in addition to RBC lysis in the presence of denatured LLO (10 nM) alone, buffer (PBS), and non-denatured LLO (10 nM). Asterisks (*) and daggers (‡) indicate statistical significance of *P* ≤ 0.05 using a One-way ANOVA with Tukey’s multiple comparisons test compared to denatured LLO alone and WT PrsA2*,* respectively and the error bars represent the standard error of the mean. Assays were performed in triplicates.

### The serine-rich C-tail of PrsA2 is important for PPIase activity

Since both the PrsA2 C-tail and serine residues within the PrsA2 C-tail are needed for efficient LLO activity (Fig 8), we asked whether other PrsA2 functional activities are also affected by these mutations. Therefore, we tested for PPIase activity of recombinant PrsA2ΔC-tail and PrsA2-StoA-tail as compared to wildtype PrsA2 using a protease-coupled assay that measures the *cis*-to-*trans* isomerization of a tetrapeptide substrate (*N*-Succinyl-Ala-Phe-Pro-Phe-*p*-Nitroanilide) [28,45,46]. For enzymes that exhibit PPIase activity, the *p*-Nitroanilide dye, linked to the tetrapeptide, is released by alpha-chymotrypsin cleavage upon the isomerization of the prolyl peptide bond resulting in a yellow color formation. We observe the rate of tetrapeptide cleavage upon the addition of wildtype PrsA2, PrsA2ΔC-tail, or PrsA2-StoA-tail recombinant proteins in Fig 9A. The PrsA2ΔC-tail mutant exhibits an overall reduction in its ability to catalyze the *cis*-to-*trans* isomerization of the tetrapeptide substrate (Fig 9A). The PrsA2-StoA-tail mutant showed an intermediate phenotype, where it was less efficient in catalyzing the *cis*-to-*trans* isomerization of the tetrapeptide substrate at some time points when compared to wildtype PrsA2 (Fig 9). Focusing on two time points, 150 seconds and 250 seconds, the PrsA2-StoA-tail mutant shows similar levels of PPIase activity to wildtype PrsA2 at the earlier time point, which is reduced at the later time point, while the PrsA2ΔC-tail is significantly less than wildtype PrsA2 at both time points (Fig 9B&C). Interestingly, although the mutations we tested are not in the predicted PrsA2 active site [28,32], these results suggest that in addition to the entire PrsA2 C-tail, the serine residues within the PrsA2 C-tail are important for efficient PrsA2 PPIase activity.

**Fig 9:**
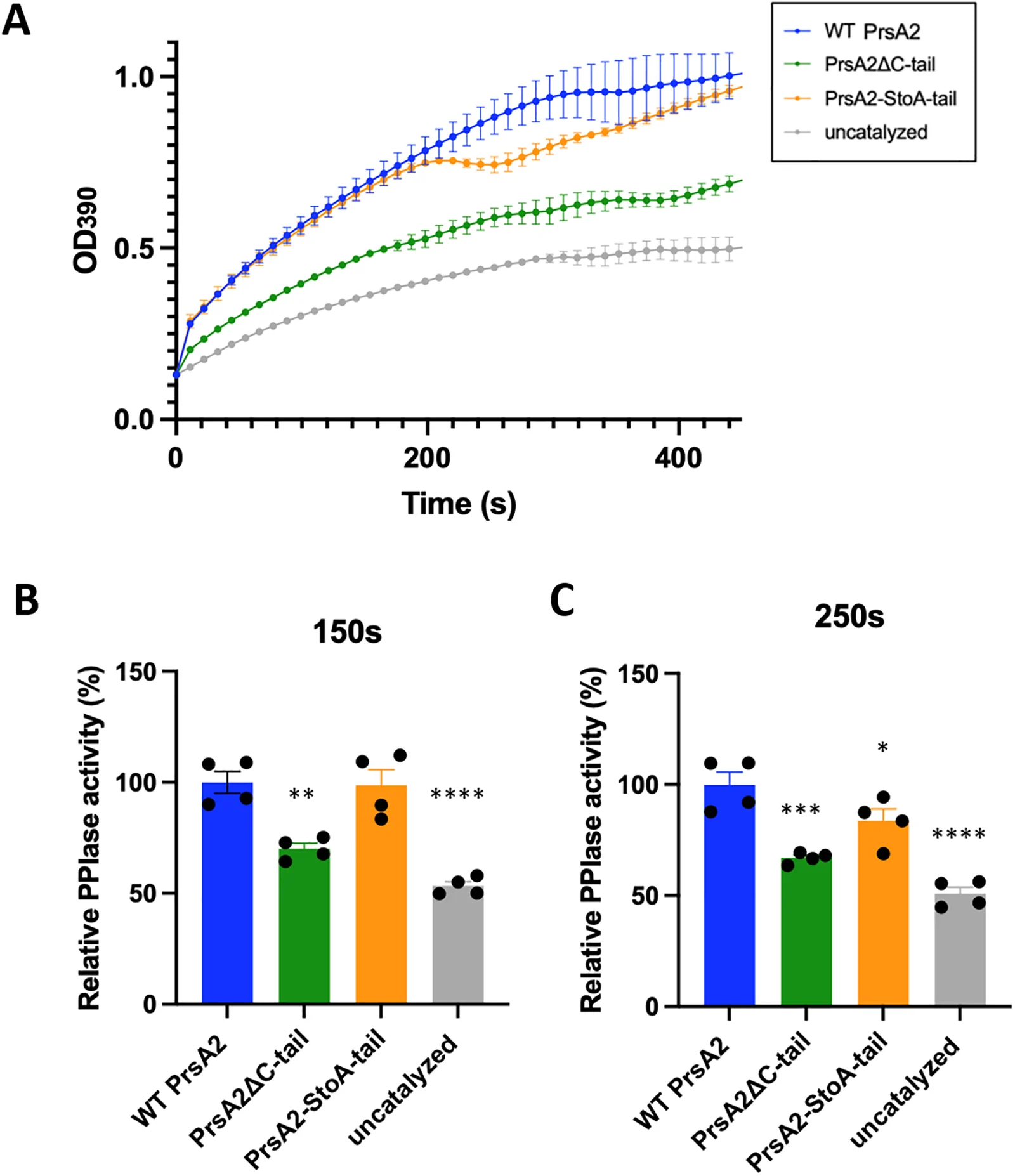
The C-tail of PrsA2 is required for functional PPIase activity. **A)** PrsA2 (8.0μM) accelerates the rate of *cis*-to-*trans* isomerization of the tetrapeptide substrate *N*-Succinyl-Ala-Phe-Pro-Phe-*p*-Nitroanilide allowing for chymotrypsin cleavage. The PrsA2ΔC-tail (8.0μM) is unable to effectively catalyze the *cis*-to-*trans* isomerization of the tetrapeptide substrate, whereas the PrsA2-StoA-tail (8.0μM) shows a slight reduction in overall activity after 200 seconds compared to WT PrsA2. **B)** The relative PPIase activities of the PrsA2 tail mutants are compared to WT PrsA2 at 150 seconds and the PrsA2ΔC-tail demonstrates reduced PPIase activity. Data was normalized to the uncatalyzed control. **C)** The relative PPIase activities of the PrsA2 tail mutants are compared to WT PrsA2 at 250 seconds and the PrsA2ΔC-tail and PrsA2-StoA-tail proteins exhibit reduced PPIase activity. Data was normalized to the uncatalyzed control. Statistical significance was determined by One-way ANOVA with Tukey’s multiple comparisons test where ****P ≤ 0.0001, ***P ≤ 0.001, **P ≤ 0.01, and *P ≤ 0.05. Error bars represent the standard error of the mean.

### PrsA2 C-tail mutants exhibit reduced swimming motility and decreased ethanol survival but demonstrate normal growth at acidic pH and at high osmotic stress

PrsA2 is a multifaceted chaperone that in addition to virulence is required for specific roles in cell physiology and stress survival [29,32]. *Lm* strains lacking *prsA2* exhibit reduced swimming motility and increased sensitivity to ethanol, extreme pH, and high osmotic stress environments [29,32]. Noting that the PrsA2 C-tail is required for virulence and functional PPIase activity, we tested this PrsA2 unstructured region for contributions to specific physiological phenotypes. First, we tested for swimming motility, where *Lm* strains are motile at lower temperatures (30°C) [47]. Strains expressing the *prsA*2-StoA-tail were similar to wildtype; however, the *prsA*2ΔC-tail mutant was defective in motility, as shown by reduced swimming colony size (Fig 10A). These data suggests that the PrsA2 C-tail is required for the maturation of a functional flagellum.

**Fig 10:**
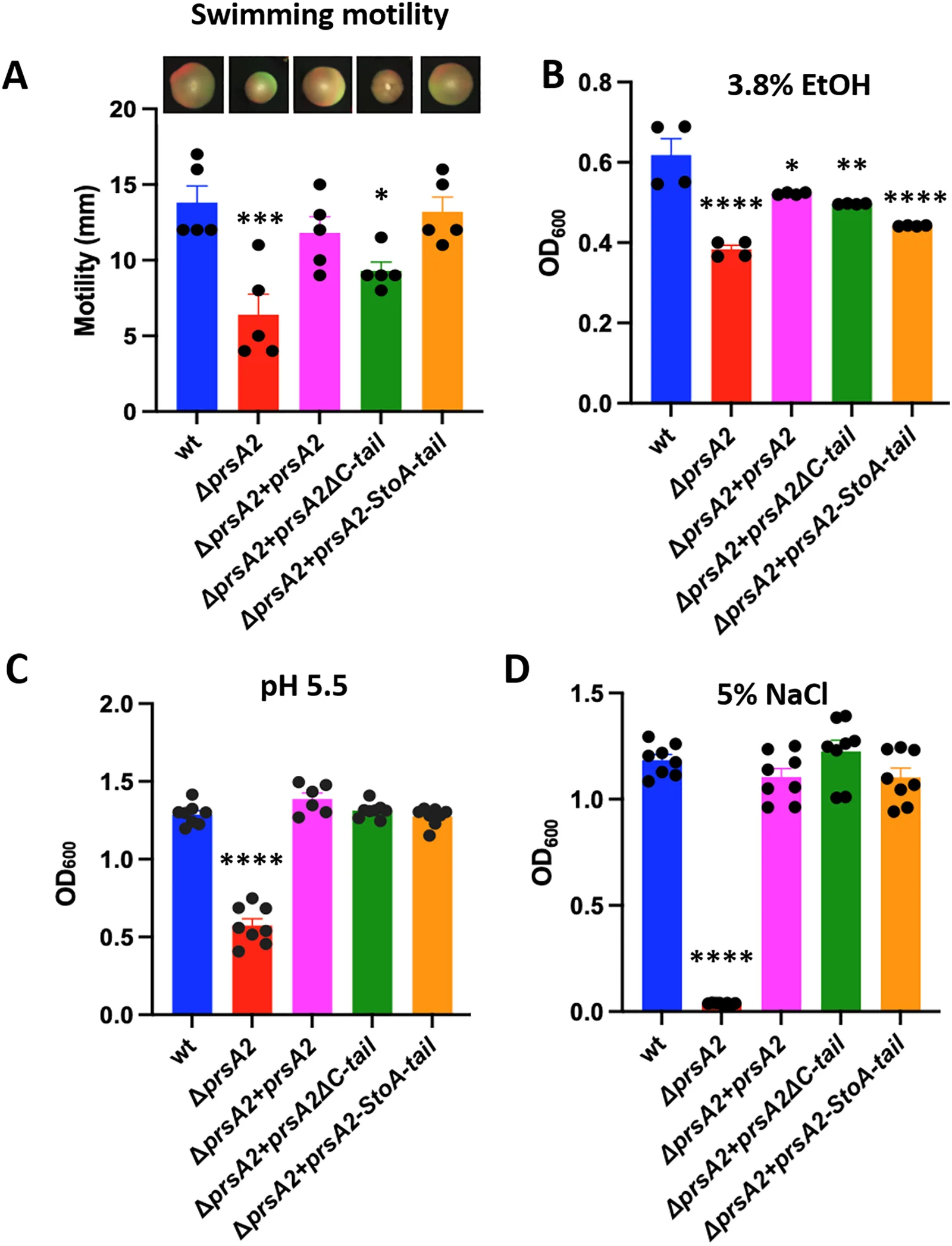
The contribution of the *Lm prsA2* C-tail to swimming motility and stress resistance. **A)** Swimming motility of *Lm prsA2* C-tail mutants. Motilities were assessed by measuring the diameters (mm) of the swimming colonies. A representative image of the swimming colonies is provided from five independent experiments. **B)** Bacterial survival in 3.8% ethanol (EtOH). Saturated bacterial cultures are inoculated into BHI broth with 3.8% EtOH and grown overnight at 37ºC with agitation. The bacterial growth is shown as optical density (OD_600_). **C)** Bacterial growth at acidic pH 5.5. Saturated bacterial cultures were inoculated into BHI broth pH 5.5 and grown overnight at 37ºC with agitation. The bacterial growth is shown as OD_600_. **D)** Bacterial growth at high osmolarity. Saturated bacterial cultures were inoculated into BHI broth supplemented with 5% NaCl and grown overnight at 37ºC with agitation. The bacterial growth is shown as OD_600_. Statistical significance was determined by a One-way ANOVA with Tukey’s multiple comparisons test compared to wildtype where ****P ≤ 0.0001, ***P ≤ 0.001, **P ≤ 0.01, and *P ≤ 0.05. Error bars represent the standard error of the mean. All BHI broth experiments were performed at least four independent times.

To determine if the C-tail of PrsA2 confers resistance to physiological stress conditions, we grew these *Lm* strains in BHI containing 3.8% ethanol (Fig 10B), or at pH 5.5 (Fig 10C) or supplemented with 5% NaCl (Fig 10D). We observe that both the PrsA2 C-tail and serine residues within the PrsA2 C-tail are required for growth in 3.8% ethanol, as strains expressing the *prsA*2ΔC-tail or the *prsA*2-StoA-tail showed a reduction in growth compared to the wildtype strain (Fig 10). Interestingly, growth at acidic pH or high osmolarity does not require the PrsA2 C-tail (Fig 10C&D), suggesting that not all PrsA2 chaperone substrates require this unstructured PrsA2 region.

### The serine-rich C-tail of PrsA2 is critical for cell wall active antibiotic resistance

*Lm* Δ*prsA*2 strains demonstrate increased sensitivity to cell wall active antibiotics and antimicrobials, including bacitracin, vancomycin, penicillin, and lysozyme [7,28,29,32]. Therefore, we asked whether the *Lm prs*A2 C-tail mutants similarly show an increased sensitivity to cell wall targeting antibiotics and antimicrobials. *Lm prs*A2 C-tail mutants were not susceptible to inhibition of lipid carrier recycling by bacitracin [48] (Fig 11A). However, in comparison to the wildtype strain, the *Lm prsA*2ΔC-tail mutant exhibits increased susceptibility to vancomycin that inhibits cell wall transglycosylation and transpeptidation reactions [49,50] (Fig 11B); in addition to penicillin that inhibits transpeptidation reactions [51] (Fig 11C). Further, the *Lm prsA*2ΔC-tail strain is more sensitive to lysozyme, an antimicrobial that catalyzes the hydrolysis of peptidoglycan linkages [52] (Fig 11D). Surprisingly, the *Lm prsA*2-StoA-tail mutant exhibits no defects when compared to the wildtype strain (Fig 11B–D), indicating that the serine residues within the PrsA2 C-tail are dispensable for resistance to vancomycin, penicillin, and lysozyme. Taken together, these results suggest that the PrsA2 C-tail contributes to cell wall physiology and integrity.

**Fig 11:**
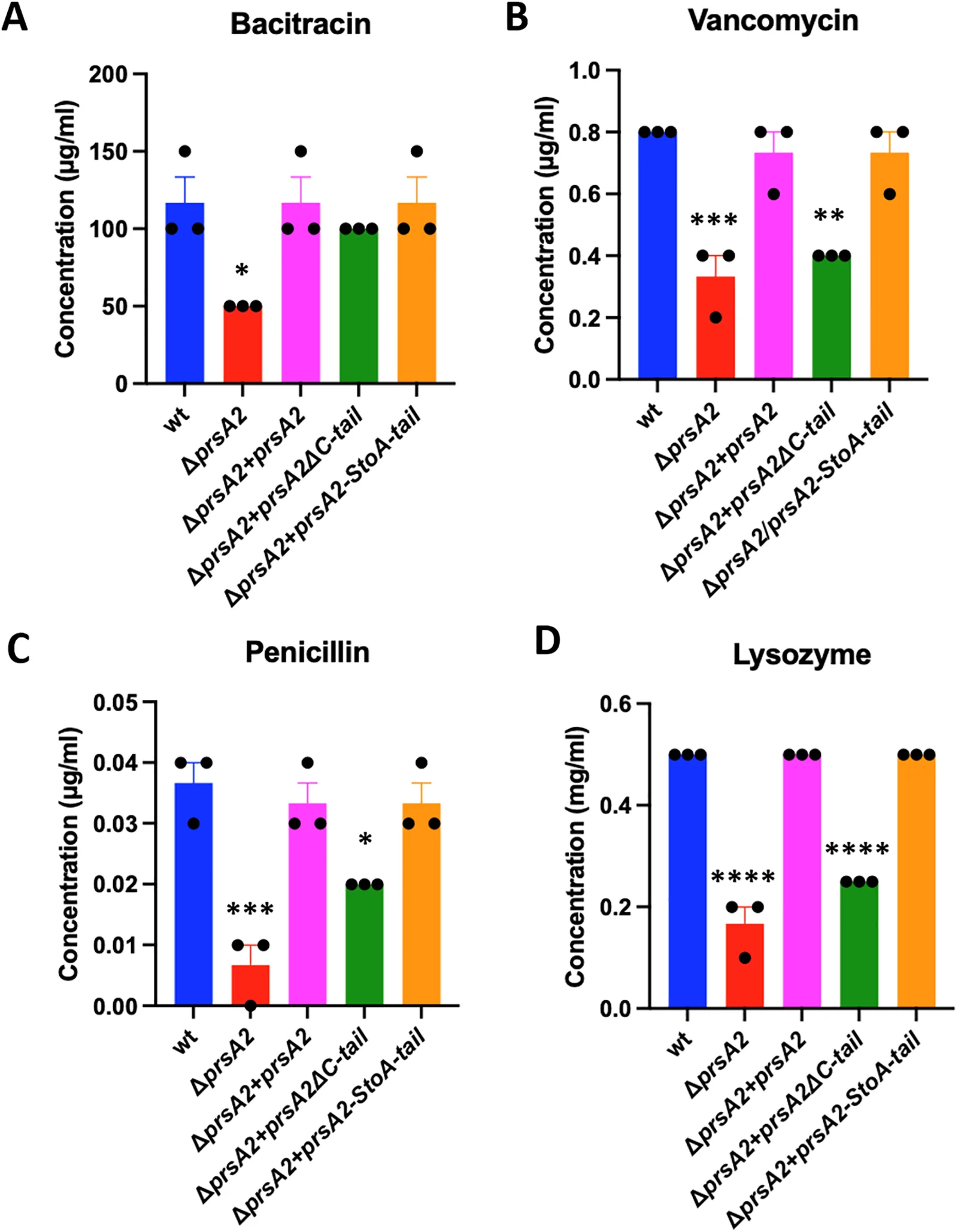
The contribution of the *Lm prsA2* C-tail to cell wall antibiotic resistance. Bacterial inoculation into broth culture containing dilutions of the cell wall active antibiotics or antimicrobials was used to determine the minimum inhibitory concentration (MIC) to prevent growth. **A)** MIC of bacitracin, an antibiotic that inhibits lipid carrier recycling [48]. **B)** MIC of vancomycin which inhibits cell wall transglycosylation and transpeptidation reactions [49,50]. **C)** MIC penicillin which inhibits cell wall transpeptidation reactions [51]. **D)** MIC of lysozyme, an antimicrobial that catalyzes the hydrolysis of peptidoglycan linkages [52]. Statistical significance was determined by a One-way ANOVA with Tukey’s multiple comparisons test compared to wildtype where ****P ≤ 0.0001, ***P ≤ 0.001, and *P ≤ 0.05. Error bars represent the standard error of the mean of 3 independent biological experiments.

## Discussion

Protein IDRs can contribute to rapid binding interactions, where the flexibility of the IDR allows the protein to adopt several conformations enabling interactions with a wide range of substrates [38]. In Gram-negative bacteria, IDRs of ATP-dependent chaperones are critical for substrate interactions, enabling the chaperone to bind to multiple substrates and assist in protein folding [53–55]. Here, we demonstrate that an unstructured region of an ATP-independent chaperone in Gram-positive bacteria is critical for bacterial virulence and physiology. In addition, we reveal that the C-terminal tail of the *Lm* secreted chaperone PrsA2 is required for the efficient interaction and folding of the key virulence factor LLO.

During infection, *Lm* depends on the secretion of virulence factors for physiology, growth, and pathogenesis [4,56]. A major *Lm* virulence factor is the cholesterol-dependent pore forming toxin, LLO, where loss of LLO activity causes a significant attenuation in bacterial virulence [26,27,57,58]. After host cell invasion, LLO forms pores in the host phagosome membrane, thereby allowing *Lm* to escape and replicate in the host cytosol [27,31,59–61]. In the absence of LLO, *Lm* remains within the host phagosome unable to escape and unable replicate to high densities, thus reducing *Lm* infectivity [27,31]. Interestingly, during *Lm* infection, the proper secretion, stability, and folding of LLO is regulated by the secretion chaperone PrsA2 [31]. This regulation occurs at both the transcriptional and translational level, as the 5′ UTR of the *prsA*2 mRNA base pairs with the 3′ UTR of the *hly* mRNA (encodes LLO) [62] and this *hly*-*prsA*2 RNA interaction is required for *Lm* virulence and pathogenicity [62]. Our work further expands on these findings, showcasing how the PrsA2 C-tail is important for bacterial virulence and secreted LLO pore forming toxin activity.

*Lm prsA2* deletion mutants are reduced in cell-to-cell spread and are attenuated in mouse virulence models [7,30,42]. Here, we reveal that both the PrsA2 C-tail and serine residues within this tail are unable to fully restore cell-to-cell spread and mouse virulence (Fig 2 & S7A). In addition, we find that the *prsA2* deletion mutant, *prsA2*ΔC-tail, and *prsA*2-StoA-tail strains exhibit prominent defects in intracellular growth compared to the wildtype strain in RAW264.7, J774, bone-marrow derived macrophages, and Caco-2 cells (Fig 3, 4, 5, S5, & S6). However, the mouse virulence defects observed in the *prsA*2ΔC-tail and *prsA*2-StoA-tail mutants was less severe than the *prsA2* deletion mutant, suggesting the C-tail serine residues retains important PrsA2 functions in the host (Fig 2). We speculate that the *prs*A2 C-tail mutants are less deficient in the maturation of secreted virulence factors than the *prsA*2 deletion mutant [5,31], as the *prs*A2 C-tail mutants produce phospholipase activity (S7B Fig). This suggests that the *prsA*2 C-tail mutants may also produce additional functional secreted virulence factors such as ActA that allows for cell-to-cell spread [11,21]. Interestingly, although the *prsA*2-StoA-tail mutant exhibited fewer defects than the *prsA*2ΔC-tail mutant in several *in vitro* assays, it displayed a greater attenuation during mouse infection (Fig 2). This discrepancy suggests that the conserved serine residues contribute to host specific PrsA2 functions that are not captured by the *in vitro* assays. We speculate that the biochemical properties of the C-tail support interactions with certain client proteins, whereas the conserved serine residues provide an additional regulatory function, potentially through post-translational modifications (PTMs), to modulate interactions with virulence-associated client proteins during infection.

Since the *prsA2*ΔC-tail and *prsA*2-StoA-tail bacterial mutants were both unable to restore secreted LLO activity (Table 1), we characterized the interaction of recombinant PrsA2 C-tail mutants with LLO. We demonstrate that recombinant PrsA2 can effectively bind and fold LLO, while the PrsA2ΔC-tail mutant had a striking ~24-fold reduction in binding affinity to LLO (Table 2). Previous AlphaFold2 models predict that PrsA2 binds to the cholesterol-binding domain of LLO, where each monomer of PrsA2 interacts with LLO in a stable confirmation through salt bridges, hydrogen bonds, and hydrophobic interactions [31]. Native PAGE analysis highlights that the PrsA2ΔC-tail mutant forms dimers, as well as higher order oligomers (S9 Fig), indicating that the reduced ITC stoichiometry is due to partial association to LLO by one PrsA2 monomer per dimer. We postulate that the C-tail may be required for initial recognition and/or stabilization of LLO, which is consistent with the thermodynamic profiles, where both the enthalpy and entropy are less favorable than the interaction of wildtype PrsA2 with LLO (Table 2). Additional members of the PrsA family, like *Bacillus subtilis* PrsA, form stable dimers in solution [63], indicating that oligomerization is a structurally conserved feature of these chaperones. In addition, since interaction between the PrsA2ΔC-tail mutant and LLO is weak, LLO may not be fully functional upon release. Moreover, the enthalpy of this interaction indicates less heat is released as compared to the wildtype PrsA2 interaction suggesting that LLO may adopt a less stable structure with more configurations, causing higher entropy (Fig 6E, S13A-B, & Table 2). To this point, we demonstrate that PrsA2ΔC-tail is defective in its ability to fold chemically denatured LLO (Fig 8 & S15).

In contrast to the recombinant PrsA2ΔC-tail mutant that demonstrates reduced binding to LLO, the PrsA2-StoA-tail mutant interacts with LLO with higher affinity than wildtype PrsA2 (Fig 6C&F, Table 2, and S14A-B Fig). We predict that the mutation of the PrsA2 C-tail serine residues to alanine allows this mutant to bind to LLO and produce a complex with higher stability that dissociates slowly. We postulate that mutation of the PrsA2 C-tail serine residues to alanine causes a loss in flexibility of this C-terminal region. While computational methods predict that the PrsA2-StoA-tail mutant maintains disorder-like characteristics, the putative disorder propensity is reduced compared to wildtype PrsA2 (S3 Fig), implying that this region of PrsA2 may be less flexible, thus leading to a more stable confirmation with potentially stronger binding to LLO. Further, mutating the PrsA2 C-tail serine residues to alanine increases the hydrophobicity of this region, and since the predicted PrsA2-LLO complex is dominated by hydrophobic interactions [31], the association of the PrsA2-StoA-tail mutant with LLO may be enhanced by more hydrophobic interactions. In addition, the thermodynamic profiles further confirm that the PrsA2-StoA-tail and LLO interaction is more favorable than the wildtype PrsA to LLO (Table 2). Interestingly, the PrsA2-StoA-tail mutant is somewhat defective in folding LLO (Fig 8 & S15). We speculate that this intermediate phenotype is caused by the inability of this mutant to effectively release LLO.

The PrsA2 C-tail is primarily composed of serine residues and a single lysine residue (Fig 1B). Protein IDRs are frequently sites of PTM enrichment, although this is not universal across all IDRs, PTMs in this context can serve diverse functional roles [36,37,64,65].. Therefore, the unstructured PrsA2 C-tail region has the potential to be targeted for phosphorylation, glycosylation, and/or acetylation. Additionally, the enrichment of serine residues at the C-terminal end of PrsA homologs is conserved among many Gram-positive bacteria (S2A&B Fig). Previous work has shown that in the Gram-positive *Streptococcus mutans*, *O*-glycosylation of the C-terminal IDR of PrsA protects the protein from proteolytic cleavage and is critical for protein-based biofilm formation under ethanol stress conditions [66]. Interestingly, *Lm* PrsA1 and PrsA2 share 75% amino acid similarity and 58% identity, and both have serine-rich C-terminal tails [29]; however, PrsA2 has several described roles in virulence and physiology while very few roles for PrsA1 have been identified. In addition, of the identified activities of PrsA1 such as resistance to oxidative stress and ethanol stress, PrsA2 is also crucial for these functions [32,42,67]. *Lm* PrsA2 is critical for several aspects of bacterial virulence such as secreted LLO activity, cell-to-cell spread, and mouse virulence [29], whereas *Lm* PrsA1 demonstrates only a minor role in transit from the intestine to distal organs [42]; therefore, we investigated PrsA2 due to its established role in virulence. Our work demonstrates that several PrsA2 activities require an unstructured C-terminal tail whereas other activities require the serine residues within the PrsA2 C-terminal tail. In the context of the host, the PrsA2 C-tail serine residues are critical for virulence (Fig 2). In addition, we demonstrate that under conditions of indiscriminate protein denaturation, such as ethanol exposure, the PrsA2 C-tail serine residues are also critical for full activity (Fig 10B). Taken together, these results support the hypothesis that the PrsA2 C-tail serine residues contribute to maintaining protein homeostasis through a potential PTM-dependent regulatory mechanism, allowing for dynamic control over PrsA2 interactions with specific client proteins under host conditions.

Strikingly, the PrsA2 C-tail serine residues are also dispensable for many PrsA2 activities. This includes swimming motility, where only the *Lm prsA2*ΔC-tail mutant was unable to restore flagellum-mediated motility (Fig 10A). Previous work identified a flagellar hook protein (Flgl) as a potential PrsA2 substrate [28], and the PrsA2 C-tail could be important for regulating Flgl as well as other flagellar motility proteins. Additionally, the PrsA2 C-tail is critical for cell wall biosynthesis, as *Lm prsA2*ΔC-tail mutants were unable to restore resistance to cell wall targeting antibiotics and antimicrobials (Fig 11B–D). Specifically related to the penicillin defect demonstrated by *prsA2*ΔC-tail mutants, a previous study showed that the localization of three PBP proteins was altered in the absence of the PrsA2 PPIase domain [28]. This suggests PrsA2 C-tail maintains cell wall integrity, potentially by enhancing the specificity between PrsA2 and certain cell wall modifying enzymes (Fig 11B–D). Recent work in *B. subtilis* demonstrates that IDRs from two secreted proteins, RsgI and PBP1, attach to the outer membrane leaflet and are critical for cell wall homeostasis [68]. However, since neither of these proteins are chaperones, the mechanism by which PrsA2 maintains cell wall integrity is likely distinct. Interestingly, we also identified stress conditions where the PrsA2 C-tail is completely dispensable which includes survival at acidic pH and high osmolarity, where both *prsA2* C-tail mutants grew similarly to the wildtype strain (Fig 10C&D). These results indicate that the PrsA2 foldase and PPIase domains are sufficient to promote growth at acidic pH and high osmolarity and indicates that not all PrsA2 activities require the C-terminal tail.

As a PPIase chaperone, PrsA2 has the ability to isomerize the tetrapeptide substrate *N*-Succinyl-Ala-Phe-Pro-Phe-*p*-Nitroanilide, accelerating its cleavage by chymotrypsin (Fig 9A). Interestingly, removing the C-tail of PrsA2 significantly reduces its PPIase activity, suggesting that this region of PrsA2 is required for functional PPIase activity (Fig 9). Since the PrsA2 C-tail does not occupy the PPIase active site, the PrsA2 C-tail may be critical for the initial interaction with the tetrapeptide, stabilization of the tetrapeptide interaction at the active site, and/or for release of the tetrapeptide from the active site. In contrast, the PrsA2-StoA-tail mutant follows the same kinetics as wildtype PrsA2 up to 200 seconds (Fig 9). We speculate that the decrease in activity after 200 seconds could indicate altered enzyme-substrate interaction dynamics. Taken together, our results demonstrate that the PrsA2 C-tail is required for tetrapeptide PPIase activity.

Protein IDRs are understudied in Gram-positive bacteria. Our work, focusing on the contributions of the unstructured, serine-rich C-tail of PrsA2 to promote *Lm* bacterial virulence and physiology provides another example of the dynamic functions of unstructured regions in bacteria. In addition, since PrsA is well conserved in Gram-positive bacteria, this work is relevant to other Gram-positive pathogens. Here, we determined that the *Lm* PrsA2 C-tail is critical for the interaction and activation of the pore forming toxin, LLO. We hypothesize that this region may be crucial for interactions with several secreted PrsA2 protein clients. Future studies on whether the PrsA2 C-tail is a target for PTMs may provide evidence on how specific clients are prioritized. In addition, future work focusing on determining the interaction pose between PrsA2 and LLO will provide further insight at a molecular level of how secretion chaperones interact with client proteins.

## Materials and methods

### Ethics statement

All animal experiments were conducted at the University of Illinois at Chicago ABSL2 facilities under Institutional Animal Care and Use Committee approved protocol #18–094, in accordance with the United States Department of Agriculture, Public Health Service, and Office of Laboratory Animal Welfare guidelines. All animal procedures complied with institutional ethical standards.

### Bacterial strains, plasmids, and media

Strains used in this study are listed in Supplemental S1 Table. The *Lm* wildtype strain is 10403S while the *Lm* Δ*prsA2* strain is 10403S containing an erythromycin resistance gene (*erm*) in place of the *prsA2* coding sequences [30]. The *Lm* Δ*prsA2* strain was used for complementation with the integration plasmid pIMK2 [69] containing *prsA2 [*29*]*, *prsA*2ΔC-tail, or *prsA*2-StoA-tail. *Escherichia coli* One Shot TOP10 cells (Invitrogen) and *E. coli* S17 cells [29] were used as host strains for recombinant plasmids. *E. coli* DE3* (Invitrogen) was used for expression of recombinant proteins from plasmids pQE60-*prsA2* [28], pET15b-*prsA*2ΔC-tail, pQE60-*prsA*2-StoA-tail, pQE30-LLO [31], pET-*prsA2*-TEV-6xHis, pET-*prsA*2ΔC-tail-TEV-6xHis, and pET-*prsA*2-StoA-tail-TEV-6xHis. Luria broth (LB) and brain heart infusion (BHI) medium were used for growth of *E. coli* and *Lm*, respectively unless otherwise specified.

### Construction of *Lm* Δ*prsA2* strains complemented with the *prsA2*ΔC-tail or the *prsA2*-StoA-tail mutations

Genomic DNA was isolated from *Lm* 10403S and used as template DNA for the amplification of the *prsA2* serine tail deletion with primers LmPrsA2NcoF1 and LmPrsA2R1ClaISerDel (Supplemental Table S2). The forward and reverse primers were designed with an NcoI and ClaI restriction site, respectively and the reverse primer was designed to remove PrsA2 amino acids 284–293. The PCR-amplified product was cloned into the expression plasmid pIMK2 that contains a constitutive promoter for target gene expression and integrates into a single neutral site within the *Lm* chromosome [69]. The previously constructed plasmid pIMK2 containing wildtype *prsA2* [29] was used for site directed mutagenesis to generate the serine to alanine PrsA2 tail mutant. Sequential site directed mutants were generated using a kit and primers designed as specified by the manufacture until all PrsA2 tail serine residues were substituted to alanine residues (QuikChange Lightning, Agilent Technologies) (Supplemental Table S2). Plasmid constructs were propagated in *E. coli* One Shot TOP10 cells, isolated by mini-prep and transformed into *E. coli* S17 cells, and subsequently introduced into the *Lm* Δ*prsA2* strain by conjugation. Constructs and strains were verified by DNA sequencing analysis.

### Growth curve of *Lm* strains

Saturated overnight bacterial cultures were diluted 1:20 in BHI broth and were grown with agitation at 180 rpm at 37°C. Bacterial growth was measured every hour for eight hours as determined by optical density (OD) measurements at 600nm (OD_600_). Experiments were conducted in triplicate, and the standard error of the mean was determined.

### Isolation of bacterial cell-associated proteins for Western blot analysis

*Lm* strains were grown overnight in BHI broth then diluted 1:20 in fresh BHI broth. Then bacterial cultures were grown for ~2.5 hours until mid-log phase (OD_600_ ~ 0.6) with agitation at 180 rpm and 37°C. To obtain cell-associated proteins, cultures were normalized to an OD_600_ of 0.6 in 50 mL volumes. Pellets were then transferred to microfuge tubes and spun at top speed. The supernatant was removed, and the pellet was resuspended in 100μl of laemmli sample buffer with beta-mercaptoethanol (BME) and boiled for 10 minutes.

### Western blot analysis

Protein samples were run on a 10% SDS-PAGE gel for 15 minutes at 100 V, then at 200 V for 1 hour. Proteins were transferred to a methanol activated polyvinylidene fluoride (PVDF) membrane at ~20 V for 1 hour. After the transfer, the membrane was blocked in PBS-T (1X PBS and 0.05% Tween) with 5% dried milk solution overnight at 4°C. The membrane was then washed with 1X PBS-T for 10 minutes and probed with polyclonal anti-PrsA2 antibody (1:2,500 dilution) [30] for 1 hour with gentle rocking. The membrane was subsequently washed twice with 1X PBS-T for 5 minutes, followed by incubation with anti-rabbit alkaline phosphatase-conjugated secondary antibody (1:2,500 dilution) for 1 hour, and then washed twice more with 1X PBS-T. Protein bands were visualized by the addition of a one-step NBT/BCIP substrate (Thermo Fisher) to the PVDF membrane for ~3 minutes and imaged using an Azure 400 Imager. To assess equal protein loading, 10 μl of cell-associated protein samples were run on a 10% SDS-PAGE gel at 200 V for 1 hour. The gel was stained with Coomassie to visualize protein loading.

### Intravenous mouse infections

Animal procedures were approved by the University of Illinois at Chicago (UIC) Animal Care Committee (ACC) protocol #18–094 and were conducted in the Biological Resources Laboratory (BRL) at UIC. Training in animal care and handling was provided by veterinary staff. Swiss Webster mice serve as a model for outbred resistance to infection and exhibit a robust immune response to *Lm* [28–30,32]. Intravenous infections were conducted as previously described [29,32]. Briefly, bacterial cultures were grown for 16 hours at 180 rpm and 37°C to saturation, diluted 1:20 in fresh BHI broth and grown to mid-log phase (OD_600_ ~ 0.6) at 37°C. Bacteria were then normalized to 6 × 10^8^ CFU (colony forming units)/mL, washed twice with PBS pH 7, diluted, and resuspended in PBS pH 7 to a final concentration of 1 × 10^5^ CFU/mL. Seven- to nine-week-old (age after one week of acclimation) female Swiss Webster mice (Charles River Laboratories) were tail vein injected with 200 μl containing 2x10^4^ CFU of bacteria. For downstream statistical analysis, 9 mice were infected per group, 45 animals in total. Tail vein injection requires brief restraint with minimal animal pain and distress. After injection, every 4–6 hours, mice were monitored for signs of distress such as fur ruffling and listlessness, animals did not exhibit signs of severe discomfort and remained for the duration of the study. Mice were sacrificed at 72 hours post infection, (one to two days prior to when death would occur following *Lm* injection [30,32]), using CO_2_ from a compressed gas cylinder followed by cervical dislocation. Then, livers and spleens of infected animals were collected, homogenized, and plated for total CFU count for each organ.

### Bacterial intracellular growth in cell culture models

RAW264.7 (mouse) macrophages, J774 (mouse) macrophages, and Caco-2 (human colon) cells were maintained as previously described [5,30,70]. The day before infection, macrophages were seeded onto 12 mm circular glass coverslips in tissue culture dishes and incubated at 37°C with 5% CO_2_, while Caco-2 cells were seeded onto coverslips and incubated for two days. Overnight cultures of *Lm* (OD_600_ ~ 0.8) were used to infect cells. RAW264.7 macrophages were infected with a multiplicity of infection (MOI) of 0.8:1, J774 macrophages were infected with an MOI of 0.3:1 and Caco-2 cells were infected with an MOI of 50:1. Macrophages were infected for 30 minutes, then washed three times with 1x PBS, followed by the addition of 10 µg/mL gentamicin at 1 hour post-infection to kill extracellular bacteria after allowing uptake into the host cells. Caco-2 cells were infected for 1 hour, then washed four times with 1x PBS, followed by 10 µg/mL gentamicin. At each indicated time point, three coverslips per strain were removed, lysed in 2 mL H2O and vortexed for 5 seconds. Serial dilutions were made in 96-well plates and lysates were plated onto LB agar plates and incubated overnight at 37°C. Then bacterial CFUs per coverslip were enumerated. The data represents two independent experiments with three replicates.

### Isolation of bone-marrow derived macrophages and intracellular growth assay

Primary bone-marrow macrophages (BMMs) were isolated as described previously [71]. Briefly, BMMs were isolated from the femurs and tibias of 5- to 7- week-old C57BL/6J mice (Jax no. 000664). Isolated progenitor cells were seeded at 5  ×  10^6^ cells in 100 mm by 26 mm petri dishes with 15 mL BMM media supplemented with 100 μg/mL Pen/Strep in a 37°C, 5% CO_2_ incubator. 10 mL of media was added to growing cells after 3 days and cells were grown for another 6 days for differentiation. For storage, adherent cells were first removed by incubation at 4^o^C in 10 mL of 1x PBS for 30 minutes and manual pipetting. Cells were pelleted at 500 x g for 5 minutes and then saved in media containing 10% DMSO at 5  ×  10^6^ cells/vial in liquid nitrogen before being used for experiments.

Bone-marrow derived (mouse) macrophages were maintained as previously described [26,72]. The day before infection, BMM were seeded onto tissue culture-treated 96-well plates and incubated at 37°C with 5% CO_2_. Overnight cultures of *Lm* (OD_600_ ~ 0.8) were used to infect cells with a MOI of 0.3:1. BMM were infected for 30 minutes, washed once with 1x PBS, and treated with bone-marrow macrophage media (BMM) containing 10 µg/mL gentamicin. At each time point, BMM was removed and cells were lysed in 90 µl H_2_O. Serial dilutions were performed in 96-well plates and plated onto LB agar and incubated overnight at 37°C. Then bacterial CFUs were counted. Data represents two independent experiments with three replicates.

### Bacterial phagocytosis of RAW264.7 macrophages

RAW264.7 macrophages were seeded onto coverslips in tissue culture plates and infected with *Lm* strains at a MOI of 0.1:1, 1:1, and 10:1. Infection proceeded for 30 minutes, then cells were washed three times with 1x PBS, followed by the addition of fresh media with 10 µg/mL gentamicin to kill extracellular bacteria. At two hours post-infection, five coverslips per strain were removed, lysed in 2 mL H_2_O and vortexed for 5 seconds. Serial dilutions were plated onto LB agar plates and incubated at 37°C overnight for enumeration of bacterial CFUs per coverslip. The data represents two independent experiments with five replicates each.

### *Lm* hemolysis assay

Hemolytic activity was measured as previously described with minor modifications [31]. Briefly, *Lm* strains were grown overnight in LB broth. These cultures were diluted (1:10) in fresh LB broth and grown for ~5.5 hours at 180 rpm at 37°C. Bacterial cells were normalized based on OD_600_. Supernatants were collected by centrifugation and serially diluted with 1 × PBS containing 1 mM DTT at pH values of 5 and 6.5, then incubated at 37°C for 30 minutes. Then, washed 20% Alsevers sheep’s RBCs (100 µL) was added to diluted supernatants, followed by incubation at 37°C for 30 min. Samples were pelleted by centrifugation at maximum speed, and the dilution resulting in 50% RBC lysis was determined. Hemolytic units are represented as the reciprocal of the dilution resulting in 50% RBC lysis. The fold reduction is represented as the hemolytic units of each mutant divided by the wildtype, and the resulting fraction was subtracted from one. Data represent 3 independent experiments with 2 technical replicates.

### *Lm* L2 plaque assays

Plaque assays were conducted as previously described [73]. Briefly, L2 fibroblasts monolayers were grown in 6-well culture dishes and infected with *Lm* at an MOI of 30:1 for 1 hour. Then, infected monolayers were washed three times with PBS pH 7 and overlaid with DMEM-agarose containing gentamicin (10 μg/mL) to kill extracellular bacteria. At 72 hours, a micrometer was used to measure plaque size.

### *Lm* phospholipase assay

Phospholipase assays were conducted as previously described [32,42]. Briefly, phospholipase activity was detected using Brilliance *Listeria* Agar containing lecithin (Oxoid). Upon *Lm* phospholipase hydrolysis of lecithin in the medium, an opaque halo is produced around the bacterial streak. Individual *Lm* colonies were struck onto the Brilliance Agar medium and incubated for 24 hours at 37°C, and then streaks were inspected for a zone of opacity surrounding the bacteria.

### Protein expression and purification

*E. coli* cultures were grown overnight in fresh LB broth supplemented with 50 µg/mL of carbenicillin for 16 hours with 180 rpm at 37°C. The overnight cultures were diluted 1:200 in 1L LB broth supplemented with 50 µg/mL of carbenicillin and grown at 37°C and 180 rpm until mid-log phase (OD_600_ ~ 0.5-0.6). At mid-log phase, recombinant PrsA2 and *PrsA2* C-tail mutants were induced by the addition of 0.8 mM isopropyl β-d-1-thiogalactopyranoside (IPTG) while LLO was induced by the addition of 0.1 mM IPTG. These 1L cultures were then grown for 12 hours at 30°C and 180 rpm. Cells were pelleted at 8,000 rpm for 45 minutes at 4°C and the pellets were frozen with liquid nitrogen and stored at −80°C for a minimum of 24 hours. For protein purification, frozen pellets were thawed on ice, resuspended in chilled lysis buffer (50 mM Tris pH 7.5, 500 mM NaCl, 25 mM imidazole), and sonicated as previously described [31]. The lysates were centrifuged at 8,000 rpm for 40 minutes at 4°C, and the supernatants were collected. The nickel bead slurry was equilibrated in a Ni-NTA column by washing twice with ddH2O and wash buffer (50mM Tris pH 7.5, 500mM NaCl, 25mM Imidazole). The supernatants were then passed through the Ni-NTA column, washed with 200 mL of wash buffer, and the proteins were eluted with 8.5 mL of elution buffer (50 mM Tris, pH 7.5, 500 mM NaCl, and 500 mM imidazole). The eluted proteins were dialyzed for 16 hours at 4°C in a dialysis buffer (20 mM 2-(N-morpholino)ethanesulfonic acid (MES), pH 6.5; 100 mM NaCl, 1 mM β-mercaptoethanol, and 10% glycerol). Dialyzed proteins were run on an SDS-PAGE gel and Coomassie stained and de-stained (50% ethanol, 10% acetic acid) for band inspection at the anticipated molecular weight (S16 Fig). Gels were imaged using an Azure 400 Imager and the protein concentrations were measured by bicinchoninic acid (BCA) assay (Pierce). Aliquots were frozen in liquid nitrogen and stored in the −80°C.

### Protein expression and purification with 6x-His tag removal

PrsA2 and PrsA2 C-tail mutants, cloned into a pET expression vector containing a tobacco etch virus (TEV) protease cleavage site followed by a C-terminal 6xHis tag (generated by VectorBuilder) were transformed into *E. coli* DE3* (Invitrogen). Then, protein expression and purification was performed as stated above and previously described [31,40], with minor modifications. After proteins were eluted with 8.5 mL of elution buffer (50 mM Tris, pH 7.5, 500 mM NaCl, and 500 mM imidazole), to cleave the 6xHis tag recombinant TEV protease (NEB) was added at a mass ratio of 1:50–1:200 (protease: protein). Proteins were dialyzed for 20 hours at 4°C in dialysis buffer. Then cleaved proteins were separated from recombinant TEV protease and un-cleaved proteins using Ni-NTA-affinity chromatography followed by adding 4 mL of dialysis buffer with 50 mM imidazole. The cleaved proteins were collected in both flow-through and in dialysis buffer with 50 mM imidazole. Proteins were run on an SDS-PAGE gel and Coomassie stained for band inspection of 6xHis tag cleavage (S17A-C Fig). A BCA assay (Pierce) was used to measure the protein concentrations and the samples were then flash frozen and stored at −80°C.

### Circular dichroism spectroscopy

Circular dichroism spectra data were collected on the JASCO *J-1500* CD *Spectrometer* at 25°C from 190-to-260 nm wavelength using a 0.2 mm path length cuvette. Recombinant protein samples were diluted in 20 mM KH_2_PO_4_–10 μM concentrations at pH 6.5. Data were normalized to the buffer and fit to a Savitzky-Golay filter. The average of three scans was collected and visualized using Spectra Manager Suite software and plotted with GraphPad Prism (v.10.1.1).

### Chemical crosslinking assay

Chemical crosslinking was done as previously described with few modifications [28,31]. Purified recombinant His-tagged PrsA2, PrsA2ΔC-tail, and PrsA2-StoA-tail mutants (500 ng/μl) were incubated with 10 µM final concentration of bis-(sulfosuccinimidyl)-substrate (BS^3^) chemical crosslinker for 1 hour at 25°C in assay buffer (20mM Hepes, 200mM NaCl at pH 6.5). Reactions were boiled for 10 minutes in 2 × SDS boiling buffer with beta-mercaptoethanol (BME) followed by separation on a 10% gel by SDS-PAGE for 1 hour. Proteins were transferred to a polyvinylidene fluoride (PVDF) membrane and probed with a polyclonal anti-PrsA2 primary antibody (1:2,500 dilution) and an anti-rabbit alkaline phosphatase-conjugated secondary antibody (1:2,500 dilution) (Thermo Fisher). The addition of a one-step NBT/BCIP substrate (Thermo Fisher) to the PVDF membrane was used for the visualization of protein bands.

### Native PAGE gel

A 10% native PAGE gel was run to assess dimer formation in purified recombinant PrsA2, PrsA2ΔC-tail, and PrsA2-StoA-tail mutants. Proteins were diluted to 3 ng and 5 ng with dialysis buffer (20 mM 2-(N-morpholino)ethanesulfonic acid (MES), pH 6.5; 100 mM NaCl, 1 mM β-mercaptoethanol, and 10% glycerol) and the reaction was added to 2X sample buffer (62.5 mM Tris-HCl, pH 6.8, 25% glycerol, 1% Bromophenol Blue) before loading. Proteins were run at 4°C at 50–70 V for up to 3 hours. The gel was subsequently stained with Coomassie to visualize protein bands.

### Protein labeling and microscale thermophoresis

NanoTemper 2nd Generation Red NHS Dye (Nanotemper Technologies) was used to label recombinant purified PrsA2, PrsA2ΔC-tail, and PrsA2-StoA-tail proteins with and without 6xHis tags. This dye is compatible with proteins that have a molecular weight larger than 5 kDa and contains a reactive NHS-ester group that binds to primary amines of protein chains to form a covalent bond. Buffer exchange (20 mM 2-(N-morpholino)ethanesulfonic acid (MES), pH 6.5; 100 mM NaCl, 1 mM β-mercaptoethanol, and 10% glycerol) was performed on proteins samples (10–30 µM) to remove incompatible buffer components. Red NHS dye (10 µg) was reconstituted with dimethyl sulfoxide (DMSO) and diluted to 300 µM with labeling buffer NHS. Proteins were then incubated in the dark at room temperature with threefold excess of 300 µM NHS dye for 45 minutes. After incubation, the labeled proteins were purified by removing the excess dye by passing the reaction through the manufacture provided gel filtration B-column. After the labeled proteins were eluted, samples were aliquoted in 15 µl volumes, flash frozen, and stored at -80°C. A BCA assay was subsequently performed to determine the new concentration of the labeled proteins, and the degree of label (DOL) was calculated using the formula *A*_650_/195,000/M/cm × concentration of labeled protein. An optimal DOL ranges between 0.5-1.0 for MST experiments.

For all MST experiments, labeled proteins (PrsA2, PrsA2ΔC-tail, and PrsA2-StoA-tail), unlabeled protein (LLO), and dialysis buffer (20 mM 2-(N-morpholino)ethanesulfonic acid (MES), pH 6.5; 100 mM NaCl, 1 mM β-mercaptoethanol, and 10% glycerol) supplemented with 0.05% Tween 20 were kept at pH 6.5. To test binding, we performed 16 serial dilutions of the unlabeled ligand (15–30 µM) with dialysis buffer in 10 µL volumes in low protein binding tubes. Labeled proteins (40–80 µM) were subsequently added to the serially diluted unlabeled ligand in 10 µL volumes and incubated for five minutes at room temperature. Samples were then loaded into 16 capillary tubes (Nanotemper Technologies), and binding was measured using the MST machine (NanoTemper Monolith NT.115). Thermophoresis was monitored at 40%–80% excitation power and 50% MST Power at 25°C with MO.control v.1.6.1. This was repeated with at least three independent replicates. All MST data were moved from the MO.control software to the PALMIST [74,75] pipeline for data analysis. Fluorescence was normalized, data points were averaged, and a 1:1 *K*_*D*_ fitting parameter was applied with a 95% confidence interval. A T-jump was used to calculate thermophoresis, and resulting *K*_*D*_ values, in the form of “*K*_*D*_ (lower limit, upper limit)”, were compared to the *K*_*D*_ values from the MO.affinity analysis software. Error bars represent the standard deviations of three or more independent replicates.

### Isothermal titration calorimetry

The thermodynamic interaction between the recombinant PrsA2, PrsA2ΔC-tail, or PrsA2-StoA-tail with LLO proteins was measured using the Affinity ITC LV machine (TA instruments) at 25°C. Frozen aliquots of recombinant protein samples previously dialyzed in ITC buffer (20 mM MES, 100 mM NaCl, 1 mM β-mercaptoethanol (BME), 10% glycerol, pH 6.5) were thawed to room temperature, centrifuged (13.3 rpm at 4°C for 20 minutes) to remove possible aggregates, and degassed under a vacuum (27/341Hg) for 15 minutes. Approximately 25- to 35-fold higher concentration of the titrants (PrsA2 and PrsA2 C-tail mutants) was injected into the titrand (LLO) for 20 injections with an injection volume of 2–2.5 µL for 300 s, stirring rate of 125 rpm, and reference power 5 µcal/s using ITCRun (v.3.8.0) (TA Instruments). Raw heat rates were corrected by averaging the final three injections and applied as an “area correction” to remove non-binding contributions prior to fitting. An independent binding model was used to fit the binding thermograms for all samples using NanoAnalyze software (v.2.2.0, TA Instruments).

### Chaperone-assisted refolding assay

The chaperone-assisted folding assay was performed as previously described with minor modification [31]. Briefly, recombinant LLO was unfolded in denaturing buffer (8 M urea, 30 mM 3-(N-morpholino) propanesulfonic acid (MOPS)-HCl, 1 mM β-mercaptoethanol, pH 6.5) for 1 hour at 25^o^C. The denatured LLO was diluted 20-fold with 1 × PBS with 1 mM DTT buffer (0.137 M NaCl, 0.0027 M KCl, 0.01 M Na_2_HPO_4_, KH_2_PO_4_, 1 mM DTT at pH 6.5) to reduce the concentration of added urea in the assay. Diluted and denatured LLO (10 nM) was then incubated in the presence of increasing concentrations of PrsA2 or PrsA2 C-tail mutants for 5 minutes at room temperature. A no chaperone sample (20-fold diluted and denatured LLO alone at a final concentration of 10 nM) and non-denatured LLO (10 nM) sample were added as internal controls. The hemolytic activity of LLO in the presence and absence of each chaperone was quantified using Alsevers sheep’s RBCs [31]. PBS-washed 5% sheep’s RBCs (125 µL) was added to the protein mixtures and incubated for 45 or 90 minutes at room temperature in round-bottomed 96-well plates. After incubation, samples were centrifuged at 1,800 rpm for 5 minutes and the amount of released hemoglobin in the supernatant fractions was quantified by absorbance at 450 nm using the Biotek Cytation5-plate reader (Agilent Technologies). A 100% RBC lysis control was generated by incubating RBCs (125 µL) with 1% Triton-X-100. The hemolytic activity was determined by measuring the amount of toxin required to lyse 50% of red blood cells. Assays were performed in triplicates for each chaperone.

### Measurement of peptidyl-prolyl isomerase activity

Measurement of peptidyl-prolyl isomerase activity has been previously described [28,45,46]. Purified recombinant His-tagged PrsA2, PrsA2ΔC-tail, or PrsA2-StoA-tail (8.0 μM) and 0.5 μl of α-Chymotrypsin (20 mg/mL) were added to assay buffer (20 mM HEPES, 140 mM NaCl, 1 mM DTT, pH 6.5) in flat-bottom 96-well plates on ice. An uncatalyzed control lacking chaperone protein was included. The proteins were incubated at -20°C for 5 minutes to allow for equilibration in the assay buffer. Subsequently, 100 μl of the tetrapeptide substrate, *N*-Succinyl-Ala-Phe-Pro-Phe-*p*-Nitroanilide (0.5 mM), was quickly added to the protein with peptide solution and gently resuspended by pipetting. Then, immediately the 96-well plate was measured at 390 nm on a Biotek Cytation 5-plate reader (Agilent Technologies). Readings were taken every 11 seconds for 10 minutes at 18°C. All assays were performed twice using three independent replicates of each chaperone protein.

### *Lm* swimming motility assay

Swimming was measured as previously described with few modifications [29,32]. Bacterial strains streaked onto fresh BHI plates were grown overnight at 37°C to obtain individual bacterial colonies. Colonies were stabbed into soft BHI agar (0.3% agar) plates and grown overnight at 30°C and then overnight at 25°C. To determine the motility of the spreading colonies, the diameters (mm) were measured. Results were obtained from 5 independent experiments.

### *Lm* growth assays with ethanol, or at high osmolarity, or acidic pH

Growth assays were performed as follows: 2 mL bacterial cultures were grown for 16 hours 180 rpm at 37°C to saturation. Then, 2 μL of each saturated culture was inoculated in fresh BHI broth containing 3.8% ethanol, 5% NaCl (w/v), or a pH of 5.5 (where NaOH was used to obtain the pH). These cultures were grown for 16 hours at 180 rpm at 37°C, and bacterial growth was measured by OD_600_. Assays were performed at least 4 independent times.

### Measurement of antibiotic and antimicrobial minimum inhibitory concentrations

The minimum inhibitory concentration (MIC) to prevent bacterial growth was determined as previously described [29,32]. Briefly, dilutions of bacitracin, vancomycin, penicillin G, or lysozyme were made into fresh BHI broth in 4 mL polypropylene test tubes. The tubes were inoculated with 2 μL of bacterial cultures at OD_600_ of 0.8 and grown at 37°C for 16 hours. The MIC was determined based on the inhibition of bacterial growth and the experiments were conducted in triplicate.

### Statistical analyses

All data were analyzed using Prism software (v.10.1.1; GraphPad Software, Inc.). Statistical tests used for the analysis of each experimental data set are indicated in the Fig legends.

## Supporting information

S1 Fig Predicted aligned error of PrsA2 Alphafold2 prediction. (TIF)

S2 Fig The serine-rich C-tail region is conserved in many PrsA orthologues. (TIF)

S3 Fig The C-tail of PrsA2 is an unstructured region. (TIF)

S4 Fig Bacterial strain growth and PrsA2 expression patterns. (TIF)

S5 Fig PrsA2 serine tail mutants are deficient in intracellular replication. (TIF)

S6 Fig PrsA2 C-tail mutations impair intracellular survival independently of initial bacterial uptake. (TIF)

S7 Fig The C-tail of PrsA2 is needed for intracellular cell-to-cell spread, but not phospholipase activity. (TIF)

S8 Fig Analysis of the secondary structure and dimer formation of recombinant PrsA2 proteins. (TIF)

S9 Fig Analysis of dimer formation *in vitro.* (TIF)

S10 Fig Interaction of LLO with PrsA2 and the PrsA2 C-tail mutants lacking 6xHis tags. (TIF)

S11 Fig ITC controls of recombinant protein and buffer. (TIF)

S12 Fig PrsA2 and LLO bind with strong nanomolar affinity. (TIF)

S13 Fig The PrsA2 C-tail of is needed for association with LLO. (TIF)

S14 Fig The PrsA2-StoA-tail is required for interaction with LLO. (TIF)

S15 Fig The C-tail of PrsA2 is critical for LLO activation. (TIF)

S16 Fig SDS-PAGE gel of purified recombinant proteins. (TIF)

S17 Fig SDS-PAGE gel of purified recombinant TEV treated proteins. (TIF)

S1 Table List of bacterial strains used in this study. (PDF)

S2 Table List of oligonucleotides used in this study. (PDF)
