# Supplementary material for: The serine-rich C-terminal tail of the Listeria monocytogenes secretion chaperone PrsA2 is critical for bacterial virulence and resistance to cell-wall active antibiotics
Source: PLoS Pathog. 2026 Oct 5;22(10):e1014676. doi: 10.1371/journal.ppat.1014676 (PMC13649129; doi:10.1371/journal.ppat.1014676)
Supplement: Supplementary file 1 [file ppat.1014676.s018.pdf]

Supplemental Table S1. List of bacterial strains used in this study.

Plasmid/Strain	Genotype	Designation	Reference
TOP10	Host strain of *E. coli* for recombinant plasmids		Invitrogen
S17	Host strain of *E. coli* used for conjugation of pIMK2 plasmids in *Lm*		[9]
LAC-153	BL21 *E. coli* for protein expression and purification of PrsA2, PrsA2 tail mutants, and LLO	DE3*	Invitrogen
NF-L100	*Lm* parent strain 10403S	wildtype	[10]
NF-L1651	*Lm* 10403S with *ΔprsA2*::*erm*	*ΔprsA2*	[10]
NF-L3632	NF-L1651 with integrated pIMK2- *prsA2*	*ΔprsA2* + *prsA2*	[9]
LAC-180	NF-L1651 with integrated pIMK2- *prsA2*ΔC-tail	*ΔprsA2* + *prsA2* ΔC-tail	This work
LAC-181	NF-L1651 with integrated pIMK2- *prsA2*-StoA-tail	*ΔprsA2* + *prsA2* StoA-tail	This work
NF-E1764	pQE60 - *prsA*2 (amino acids 21-293)	PrsA2-6-his	[11]
LAC-182	pET15b - *prsA*2ΔC-tail (amino acids 21-283)	PrsA2ΔC-tail	This work
LAC-183	pQE60 - *prsA*2-StoA-tail (starting at amino acid 21)	PrsA2-StoA-tail	This work
LAC-152	pQE30 - LLO (amino acids 25-529)	LLO-6-His	[12]
LAC-600	pET - *prsA*2 (amino acids 21-293)-TEV-6xHis	PrsA2-Δ6xHis-tag	This work
LAC-601	pET- *prsA*2ΔC-tail (amino acids 21-283)-TEV-6xHis	PrsA2ΔC-tail-Δ6xHis-tag	This work
LAC-602	pET- *prsA*2-StoA-tail (starting at amino acid 21)-TEV-6xHis	PrsA2-StoA-tail-Δ6xHis-tag	This work
