# Supplementary material for: The serine-rich C-terminal tail of the Listeria monocytogenes secretion chaperone PrsA2 is critical for bacterial virulence and resistance to cell-wall active antibiotics
Source: PLoS Pathog. 2026 Oct 5;22(10):e1014676. doi: 10.1371/journal.ppat.1014676 (PMC13649129; doi:10.1371/journal.ppat.1014676)
Supplement: Supplementary file 2 [file ppat.1014676.s019.pdf]

Supplementary Table S2. List of oligonucleotides used in this study.

Oligonucleotide	Sequence	Use
**LmPrsA2NcoF1**	5'agtaCCATGGTCCAACAACTAAGGAGTGTG	Forward primer for generation of *Lm prsA2* serine tail deletion mutant
**LmPrsA2R1ClaISerDel**	5'agtaATCGATTTATTTACCATCAAAATCTTTAAAAGC	Reverse primer for generation of *Lm prsA2* serine tail deletion mutant
**LmPrsA2F1**	5'ATCGAAAGTGCAGAACGTGG	Sequencing of *Lm prsA2*
**LmPrsA2F1RC**	5'CCACGTTCTGCACTTTCGAT	Sequencing of *Lm prsA2*
**LmPrsA2F2**	5'CTGACACTAGCGACAAAACAC	Sequencing of *Lm prsA2*
**LmPrsA2F2RC**	5'GTGTTTTGTCGCTAGTGTCAG	Sequencing of *Lm prsA2*
**SdSertoAlaF1**	5'GCTTTTAAAGATTTTGATGGTgCTTCATCCAGTGATAGCGATTC	Sequential site directed mutagenesis primer used to generate serine to alanine PrsA2 tail mutant
**SdSertoAlaR1**	5'GAATCGCTATCACTGGATGAAGcACCATCAAAATCTTTAAAAGC	Sequential site directed mutagenesis primer used to generate serine to alanine PrsA2 tail mutant
**SdSertoAlaF2**	5'GCTTTTAAAGATTTTGATGGTgCTgCATCCAGTGATAGCGATTC	Sequential site directed mutagenesis primer used to generate serine to alanine PrsA2 tail mutant
**SdSertoAlaR2**	5'GAATCGCTATCACTGGATGcAGcACCATCAAAATCTTTAAAAGC	Sequential site directed mutagenesis primer used to generate serine to alanine PrsA2 tail mutant
**SdSertoAlaF3**	5'GCTTTTAAAGATTTTGATGGTgCTgCAgCCAGTGATAGCGATTCATC	Sequential site directed mutagenesis primer used to generate serine to alanine PrsA2 tail mutant
**SdSertoAlaR3**	5'GATGAATCGCTATCACTGGcTGcAGcACCATCAAAATCTTTAAAAGC	Sequential site directed mutagenesis primer used to generate serine to alanine PrsA2 tail mutant
**SdSertoAlaF4**	5'AAAGATTTTGATGGTgCTgCAgCCgcTGATAGCGATTCATCTAAAT	Sequential site directed mutagenesis primer used to generate serine to alanine PrsA2 tail mutant
**SdSertoAlaR4**	5'ATTTAGATGAATCGCTATCAgcGGcTGcAGcACCATCAAAATCTTT	Sequential site directed mutagenesis primer used to generate serine to alanine PrsA2 tail mutant
**SdSertoAlaF6**	5'GATGGTgCTgCAgCCgcTGATgcCGATTCATCTAAATAAGCACT	Sequential site directed mutagenesis primer used to generate serine to alanine PrsA2 tail mutant
**SdSertoAlaR6**	5'AGTGCTTATTTAGATGAATCGgcATCAgcGGcTGcAGcACCATC	Sequential site directed mutagenesis primer used to generate serine to alanine PrsA2 tail mutant
**SdSertoAlaF8**	5'GATGGTgCTgCAgCCgcTGATgcCGATgCATCTAAATAAGCACT	Sequential site directed mutagenesis primer used to generate serine to alanine PrsA2 tail mutant
**SdSertoAlaR8**	5'AGTGCTTATTTAGATGcATCGgcATCAgcGGcTGcAGcACCATC	Sequential site directed mutagenesis primer used to generate serine to alanine PrsA2 tail mutant
**SdSertoAlaF9**	5'GATGGTgCTgCAgCCgcTGATgcCGATgCAgCTAAATAAGCACT	Sequential site directed mutagenesis primer used to generate serine to alanine PrsA2 tail mutant
**SdSertoAlaR9**	5'AGTGCTTATTTAGcTGcATCGgcATCAgcGGcTGcAGcACCATC	Sequential site directed mutagenesis primer used to generate serine to alanine PrsA2 tail mutant
**PrsA2-6hisA (NcoI)**	5'GCG-CCATGG-CC-TGCGGTGGCGGCGGAGAT	Forward primer for generation of *Lm prsA2* serine to alanine mutant expression construct
**PrsA2-6hisSerA (BglII)**	5'GCG-AGATCTTTTAGcTGcATCGgcATC	Reverse primer for generation of *Lm prsA2* serine to alanine mutant expression construct
